# Inkjet Printing of Drugs into Surface-Embedded Micro-Reservoirs for Drug-Releasing Implants: Influence of Solvent Properties on Deposition Behavior

**DOI:** 10.3390/jfb17080420

**Published:** 2026-08-20

**Authors:** Robert Mau, Georg Schnell, Paul Oldorf, Hermann Seitz

**Affiliations:** 1 Microfluidics, Faculty of Mechanical Engineering and Marine Technology, University of Rostock, Justus-von-Liebig-Weg 6, 18059 Rostock, Germany; robert.mau@uni-rostock.de (R.M.); georg.schnell@uni-rostock.de (G.S.); 2 Schweißtechnische Lehr- und Versuchsanstalt (SLV) Mecklenburg-Vorpommern GmbH, Alter Hafen Süd 4, 18069 Rostock, Germany; oldorf@slv-rostock.de; 3 Department Life, Light and Matter, Interdisciplinary Faculty, University of Rostock, Albert-Einstein-Str. 25, 18059 Rostock, Germany

**Keywords:** pharmaceutical inkjet printing, localized drug coating, implant surfaces, drug deposition, micro-reservoirs, crystallization, coffee-ring effect, creeping

## Abstract

**Background:** Micro-reservoirs in implant surfaces represent a promising drug carrier concept for drug delivery systems. For drug loading, inkjet printing enables highly precise droplet positioning. However, droplet drying influences drug crystallization from printed drug solution. This study investigates how evaporation-driven phenomena affect the precision and homogeneity of inkjet-based deposition of a crystallizing drug into exemplary micro-reservoirs. The aim is to guide the selection of suitable solvents and inkjet process parameters. **Methods:** Laser-drilled micro-reservoirs were fabricated as blind holes with entrance diameters of 100 µm and 400 µm in the surface of specimens of EN 1.4404 (equivalent to AISI 316L) stainless steel, a commonly used biomaterial. The reservoirs were loaded with two different drug solutions using piezoelectric drop-on-demand inkjet printing. Acetylsalicylic acid (ASA) was applied as a model drug representing crystallizing small-molecule drugs. Solvents with markedly different evaporation rates, ethanol (EtOH) as a representative high-volatility solvent and dimethyl sulfoxide (DMSO) as a representative low-volatility solvent, were selected. The number of jetted droplets per dispensing step was varied. Precision and homogeneity of the drug deposition were investigated using light and laser scanning microscopy. **Results:** Over the course of droplet drying, two phenomena, the coffee-ring effect and creeping, can impair drug deposition quality. The coffee-ring effect leads to inhomogeneous, ring-shaped drug deposits. Creeping is the evaporation-driven spreading of crystalline structures and reduces the precision of drug deposition. The EtOH-based ASA solution (c = 10 g/L) was intensely affected by both phenomena. Inhomogeneities could be partially compensated via tailoring the droplet count per dispensing step. The DMSO-based solution (c = 100 g/L) exhibited a more compact crystallization of ASA (requiring ~20% less volume in an exemplary experiment), no coffee-ring effect, and only minor creeping. **Conclusions:** The DMSO-based ASA solution enabled a more precise and homogeneous drug deposition than the EtOH-based solution under the investigated printing and crystallization conditions. EtOH-related limitations could be counteracted by controlling the number of jetted droplets per dispensing step.

## 1. Introduction

Drop-on-demand (DOD) inkjet printing is an established technology for dispensing single-picoliter droplets and is highly relevant for localized drug coating applications, such as microneedles and drug-loaded micro-reservoirs [1]. Micro-reservoirs can be defined as microscale cavities embedded in large numbers in implant surfaces. Metallic implant surfaces represent a particularly relevant application area [2,3,4].

Compared with conventional drug-carrying coatings, micro-reservoirs can offer distinct advantages. They allow high amounts of drugs [5], incorporation of different drugs into individual reservoirs [1], unidirectional, multi-stage, and time-delayed drug release [6], and improved resistance to abrasion due to embedding the drug coating in the surface [4]. Furthermore, polymeric carrier content can be minimized or eliminated. Polymeric drug carriers typically offer effective drug stabilization and high tunability of drug release, but the absence of polymeric content may provide clinical benefits by reducing adverse inflammatory responses and toxicity [7,8]. An example is the reservoir-based drug-eluting stent, as shown in [9]. It was developed as an alternative to polymer-coated drug-eluting stents in order to avoid chronic inflammations [10,11,12] or further complications such as webbing, delamination, and cracking [9,13,14]. Similar limitations of polymeric coatings are known to occur in dental and orthopedic implants [15].

Inkjet printing is governed by three key phases affecting print quality and, consequently, reservoir loading [16,17,18]:

- Droplet generation,
- Droplet impact,
- Droplet solidification (including droplet drying).

Droplet solidification is particularly important because it determines the final distribution and morphology of deposited material [1,19]. In many applications, particularly in the absence of polymers, droplet solidification is based on droplet drying through solvent evaporation. During drying, internal transport phenomena may induce the coffee-ring effect, where evaporation-driven capillary flow transports solute toward the droplet edge [16,19,20,21]. Another phenomenon, particularly relevant to drug crystallization, is creeping. It is defined as the evaporation-driven extension of crystals from the boundary of a solution onto a solid nonporous substrate [22]. During droplet drying, the crystallizing substance typically forms widespread deposits that propagate beyond the area initially wetted by the droplet on the substrate [22,23,24,25]. Creeping can be categorized as either primary or secondary [23,25]. Primary creeping is the growth of crystals directly on a substrate’s surface. Crystalline structures precipitate at the outer edge of the droplet on the substrate’s surface and expand laterally around the initially wetted area. Secondary creeping follows primary creeping and is reported to appear on previously deposited crystals, leading to more spatial expansion.

Generally, crystal growth is complex and influenced by multiple factors including the crystallization behavior of the solute [26], the type of solvent and solvent–solute interactions [26,27,28,29], the purity of the solution [30], the use of additives [25], the solubility of the substance and its concentration in the solution [19,26], as well as ambient humidity and temperature [23,26]. In particular, the evaporation rate of the solvent is considered to have a significant influence on drug formulation in inkjet printing of pharmaceuticals [18,26,31,32]. A comparison of different solvent systems exhibiting varying evaporation behaviors and their influence on local drug deposition appears highly relevant. For comparison purposes, solvent volatility can be quantified through the dimensionless evaporation number [33,34,35]. It is calculated as the ratio of the evaporation time of a given liquid volume to that of the same volume of diethyl ether. A low evaporation number (<10) indicates a highly volatile solvent, whereas a high number (>50) signifies low volatility.

The present work examines the influence of evaporation-driven phenomena on the precision and homogeneity of inkjet-based deposition of a crystallizing drug into micro-reservoirs, with emphasis on solvent selection for the drug solution to be inkjet-printed. For instance, ensuring inkjet printability may require the use of specific solvents and concentration ranges [36]. In this context, there is a lack of knowledge regarding the effects of different drug deposition characteristics resulting from drug solutions based on different solvent systems. In particular, variations in evaporation behavior are assumed to play an important role. The results present merit for consideration in selecting a suitable solvent and in determining inkjet process parameters for localized drug deposition and drug coating applications, particularly for polymer-free drug loading of implant surfaces.

The experimental setup aims for basic insights using a model system, rather than to replicate a specific implant or end-use application. It is intended to highlight trends rather than establish quantitative relationships. Ethanol (EtOH) and dimethyl sulfoxide (DMSO), two solvents commonly used in pharmaceutical inks [1], are investigated. EtOH (evaporation number of 8.3 [34]) serves as a high-volatility solvent, whereas DMSO (evaporation number of ≈700 [34]) represents a low-volatility solvent. Drug deposition is studied on micro-reservoirs (diameters of 100 µm and 400 µm). The reservoirs are laser-drilled in a surface of EN 1.4404 (also known as X2CrNiMo17-12-2, equivalent to AISI 316L) stainless steel, an established biomaterial [37,38]. The reservoirs hold relevance, for instance, for dental [4] and orthopedic implants [3], as well as reservoir-based drug-eluting stents [2]. Laser micromachining is employed because of its strong relevance for precise surface structuring of metallic implant materials [39,40]. Acetylsalicylic acid (ASA) is selected as a small-molecule model drug (molecular weight of 180.16 g/mol [41]). Small-molecule drugs are often used in pharmaceutical inkjet printing [1,17] and play a significant role in implant coating applications, particularly as anti-inflammatory, antiproliferative, and antibiotic agents [15,42]. Their low molecular weight (<1000 g/mol [43], many <550 g/mol [44]) is generally associated with a high tendency toward crystallization, particularly when there also is low structural complexity [45,46]. ASA has previously been used as a model drug in crystallization studies because it is a prominent representative of aromatic carboxylic acids [30,47]. It contains both an aromatic ring and a carboxyl group, which are found in many organic compounds, particularly anti-inflammatory and analgesic drugs. Implant coating applications utilizing ASA and other drugs based on aromatic carboxylic acids have already been demonstrated [48,49,50]. In addition, ASA shows high solubility (≥100 g/L, T = 23 °C) in both EtOH and DMSO [51].

In previous work, we identified suitable formulations and inkjet printing parameters for adequate droplet generation and impact when using EtOH- and DMSO-based ASA solutions [36], whereas the present study focuses on droplet drying. It is expected that effects such as the coffee-ring effect and creeping occur with varying intensities depending on the solvent used, thereby significantly influencing the precision and homogeneity of drug deposition. Furthermore, the influence of the number of jetted droplets per dispensing step is examined. Since this parameter directly determines the drug solution volume loaded into the reservoir, affecting the extent of droplet spreading, it is expected to be useful for tailoring drug deposition.

## 2. Materials and Methods

### 2.1. Laser Drilling of Micro-Reservoirs

For drug-loading investigations, two types of laser-drilled micro-reservoirs were used. The first type, referred to as 10050, had nominal dimensions of 100 µm in diameter and 50 µm in depth. The second type, referred to as 40050, had nominal dimensions of 400 µm in diameter and 50 µm in depth. The reservoirs were laser-drilled into steel discs using ultrashort-pulse laser ablation. The discs were made of EN 1.4404 stainless steel (NiroStyle GmbH, Ludwigslust, Germany), also known as X2CrNiMo17-12-2 and equivalent to AISI 316L stainless steel. They featured a polished surface with an arithmetic mean roughness of Ra ≈ 0.1 µm and dimensions of 6 mm in diameter and 1 mm in thickness.

Laser drilling of reservoirs of type 10050 was performed using an ultrashort-pulse laser of type TruMicro 5X50 (TRUMPF GmbH & Co. KG, Ditzingen, Germany) with a wavelength of 343 nm and a pulse duration of 6 ps. For laser drilling of the reservoirs of type 40050, an ultrashort-pulse laser of type UFFL_60_200_1030_350_SHG (Active Fiber Systems (AFS) GmbH, Jena, Germany) with a wavelength of 515 nm and a pulse duration of 300 fs was used.

After laser processing, the steel discs were cleaned for 15 min in an ultrasonic bath to remove processing residues. The neutral cleaning agent Elma Clean 65 (Elma Schmidbauer GmbH, Singen, Germany) in aqueous solution (4 parts water and 1 part Elma Clean 65) was applied. Afterwards, the morphology of the reservoirs was analyzed by confocal laser scanning microscopy (CLSM) using a LEXT OLS4000 microscope equipped with a 50×/0.95 air objective (objective lens magnification/numerical aperture) and the accompanying OLS4000 software (Version 2.2.3). Entrance diameter and volume were measured (*n* = 20). All the CLSM equipment and software were obtained from Olympus (Tokyo, Japan).

### 2.2. Preparation of Drug Solutions

Two different drug solutions were prepared for drug deposition experiments, based on preliminary investigations reported in [36]. In these preliminary studies, the inkjet printability of the model drug ASA dissolved in DMSO and EtOH at different concentrations was systematically evaluated. The objective was to identify suitable drug solutions with the highest possible drug loading that still exhibit excellent inkjet printability with the selected inkjet system.

Based on these preliminary investigations, two suitable formulations were identified. The first drug solution, denoted DMSO100ASA, consisted of ASA (≥99.0%; CAS No.: 50-78-2; Merck KGaA, Darmstadt, Germany) dissolved in DMSO (≥99.7%; Merck KGaA, Darmstadt, Germany) at a concentration of c = 100 g/L. The second drug solution, denoted EtOH10ASA, consisted of ASA dissolved in EtOH (≥99.8%; Carl Roth GmbH + Co. KG, Karlsruhe, Germany) at a concentration of c = 10 g/L. Thus, the preliminary studies resulted in two solutions with different concentrations, where the concentration of EtOH10ASA had to be lower than that of DMSO100ASA to ensure inkjet printability. An ethanol-based drug solution with an ASA concentration of c = 100 g/L (EtOH100ASA) is not a feasible option for drug deposition into micro-reservoirs due to insufficient printability [36]. It should be noted that the significant difference in drug concentration between EtOH10ASA and DMSO100ASA is suboptimal for comparative investigations of evaporation-driven drug deposition. However, it is necessary to ensure reliable inkjet printing. In line with good scientific practice, it was ensured that EtOH10ASA shows drug deposition behavior generally similar to that of EtOH100ASA. Please see Figures S20 and S21 of File S2 of the Supplementary Materials.

For the present study, the drug solutions were prepared by dissolving the drug in DMSO or EtOH under stirring at ambient temperature (T = 21 ± 2 °C) until clear solutions were obtained. According to [36], DMSO100ASA exhibits a surface tension of 42.14 ± 0.44 mN/m and a dynamic viscosity of 2.42 ± 0.06 mPa·s. In contrast, EtOH10ASA features a significantly lower surface tension of 22.32 ± 0.10 mN/m and a dynamic viscosity of 1.13 ± 0.01 mPa·s. Moreover, the wetting behavior of EtOH10ASA and DMSO100ASA on the steel surface was assessed by static contact angle measurements (*n* = 3) via sessile drop method using a droplet volume of 6 µL. The measurements were carried out utilizing the contact angle and contour analysis system OCA 40 Micro (DataPhysics Instruments GmbH, Filderstadt, Germany).

### 2.3. Drug Deposition into Micro-Reservoirs

**Figure 1:**
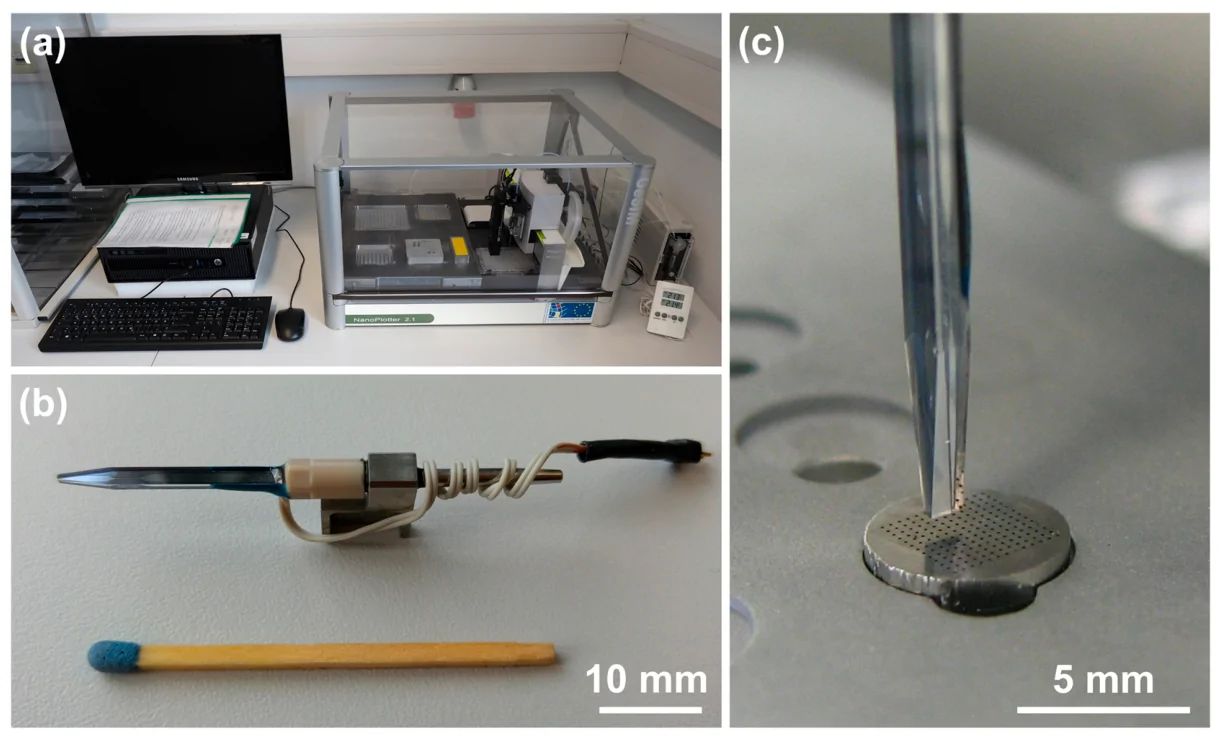
(**a**) Nano-Plotter 2.1 device for microdosing approaches. (**b**) Piezo-driven drop-on-demand inkjet printhead of type Pico-Tip J. A common matchstick is shown for size comparison. (**c**) The printhead is positioned over a laser-drilled steel disc for drug loading of reservoirs.

Drug deposition into the micro-reservoirs was performed using the drop-on-demand inkjet printing device Nano-Plotter 2.1, equipped with a piezo-driven printhead of type Pico-Tip J (both GeSiM, Gesellschaft für Silizium-Mikrosysteme mbH, Radeberg, Germany). Operation of the Nano-Plotter 2.1 device is fully automated, including printhead washing, printhead priming, camera-based target recognition, and printhead positioning with a repetition accuracy of ±10 µm. A nominal printhead-to-substrate distance of 200 µm was applied. The setup is shown in Figure 1.

In this study, both the drug solutions EtOH10ASA and DMSO100ASA were dispensed with a nominal volume of 50 pl per single droplet, containing 0.5 ng (0.0005 µg) of the model drug ASA when applying EtOH10ASA, and 5 ng (0.005 µg) when applying DMSO100ASA. Printhead parameters and washing cycles for a reproducible droplet formation were evaluated in a prior study [36]. Voltages of U = 47 V and U = 57 V were used for EtOH10ASA and DMSO100ASA, respectively. Both drug solutions were printed using a pulse duration of t_pulse_ = 20 µs and a frequency of f = 100 Hz. During the application of EtOH10ASA, the printhead was washed with water for 30 s every 4 min to prevent nozzle clogging. For DMSO100ASA, no washing procedure was needed.

An overview of the experiments performed is given in Table 1. The micro-reservoirs were loaded with different masses of the drug ASA. For reservoir type 10050, three replicate experiments were conducted, whereas for reservoir type 40050, five replicate experiments were performed. For each target drug mass, a specific number of droplets of the respective drug solution was deposited into a single reservoir. Owing to the different concentrations of the two drug solutions, the total number of dispensed droplets differs by a factor of ten. To avoid an overflow of the reservoir, the drug deposition was divided into multiple dispensing steps. For each dispensing step, a specific number of single droplets was dispensed into the targeted reservoir. Each subsequent dispensing step was applied only after the previously dispensed droplets had dried for a specific amount of time. Table 2 provides an overview of the applied drying times used in relation to the drug solution and the droplet count applied per dispensing step. The drying times were chosen to allow the droplets to dry sufficiently, thereby freeing enough reservoir volume for the subsequent dispensing step. Complete drying aimed at eliminating residual solvent was not intended.

Afterwards, the precision and homogeneity of the drug deposition were investigated via light microscopy and CLSM using the LEXT OLS4000 (50×/0.95 air objective). In terms of spatial confinement, drug deposition is considered maximally precise if the drug was deposited exclusively within the reservoir. Homogeneity is present when the drug is uniformly distributed within the reservoir. Since this work is not aimed at a specific implant application but rather is designed as an exploratory feasibility study using a simplified model system, the data were evaluated primarily in a qualitative manner. To strengthen the assessment, multiple microscopy images from replicate experiments were evaluated and assessed for limitations in both precision and homogeneity. Moreover, a comparative analysis of the drug-loaded reservoir volume was performed using CLSM data obtained from representative successful drug-loading experiments. All experiments were carried out at ambient conditions (air, T = 21 ± 2 °C, relative humidity of 50 ± 10%).

## 3. Results

### 3.1. Laser-Drilled Micro-Reservoirs

**Figure 2:**
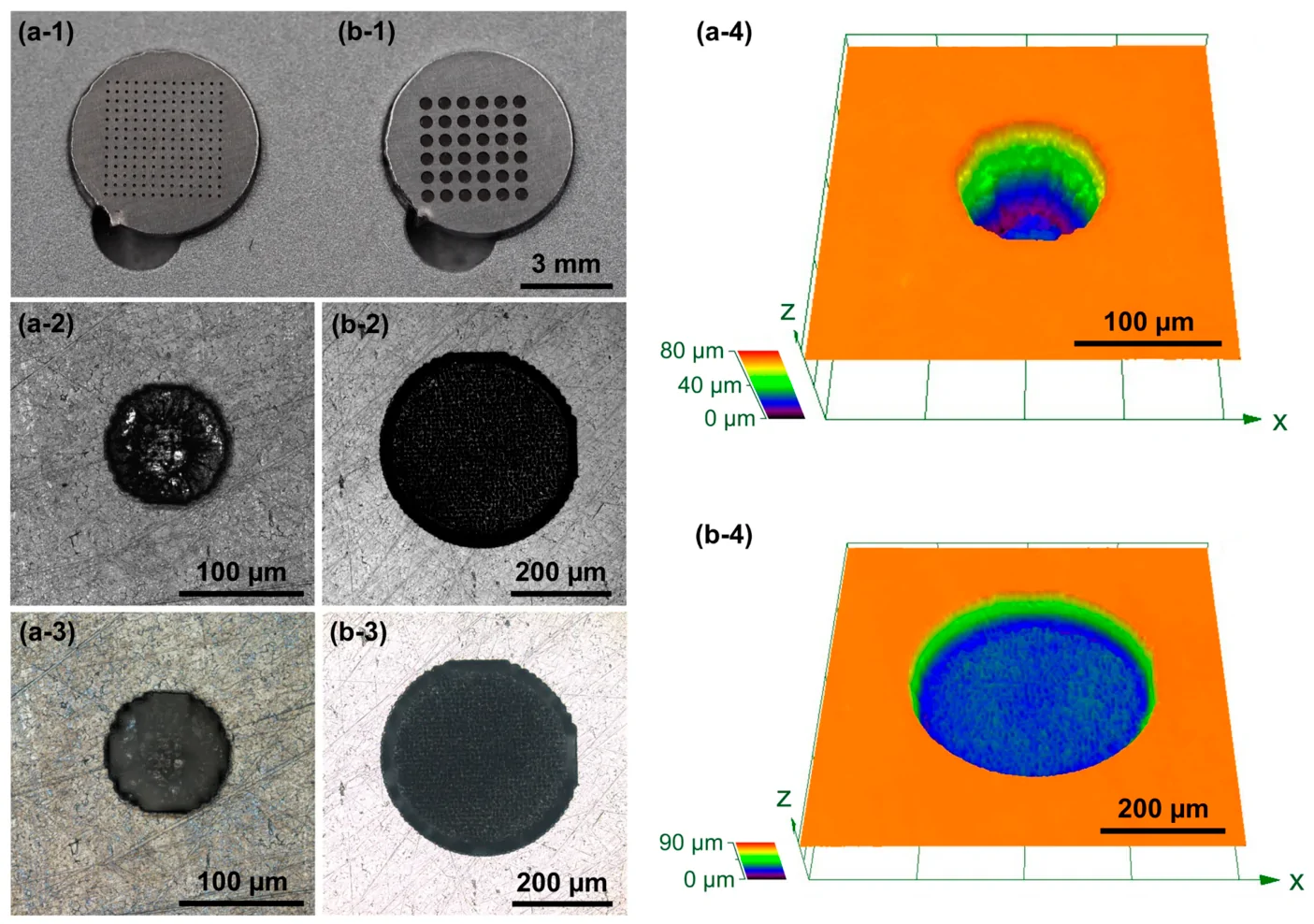
(**a-1**) Photography of stainless-steel discs with laser-drilled reservoirs of 100 µm in diameter (denoted as 10050), and (**b-1**) with laser-drilled reservoirs of 400 µm in diameter (denoted as 40050). (**a-2**) CLSM image, (**a-3**) light microscopy image, and (**a-4**) three-dimensional imaging of an exemplary, empty single reservoir of type 10050. (**b-2**–**b-4**) Corresponding images for a reservoir of type 40050, showing sharp-edged reservoirs without significant recast layers or burr formation but uneven bottoms.

Both reservoir types, 10050 and 40050, were successfully fabricated by ultrashort-pulse laser ablation, demonstrating the suitability of this process for producing well-defined micro-reservoir structures. Generally, the reservoirs are sharp-edged, without significant presence of recast layers or burr formation. The shape of the opening appears almost perfectly circular (Figure 2), and the measured diameters show high reproducibility (Table 3).

**Figure 3:**
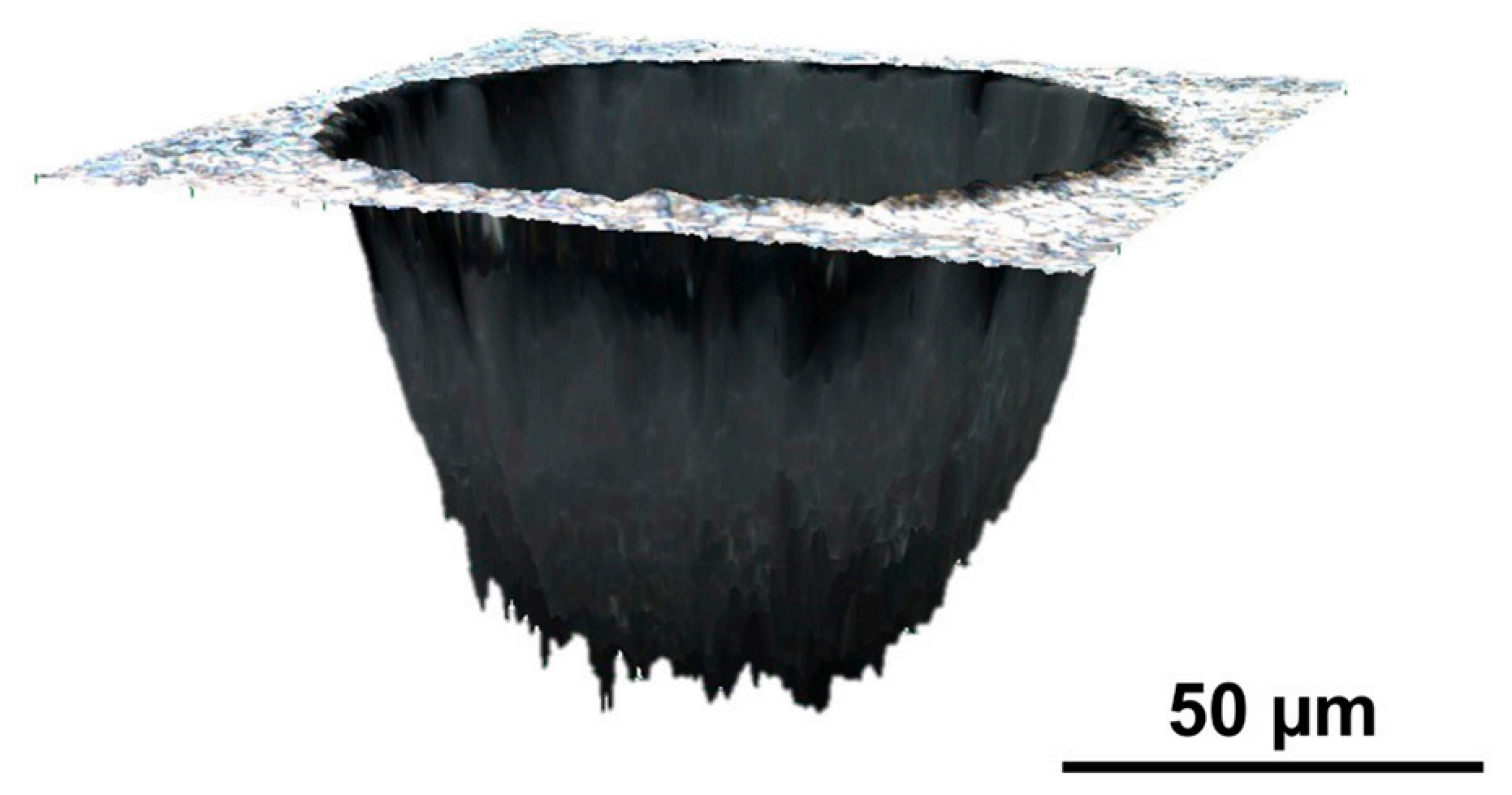
Three-dimensional imaging, featuring a more profile-like perspective, of a reservoir type 10050. The reservoirs exhibit a cone-like geometry.

**Figure 4:**
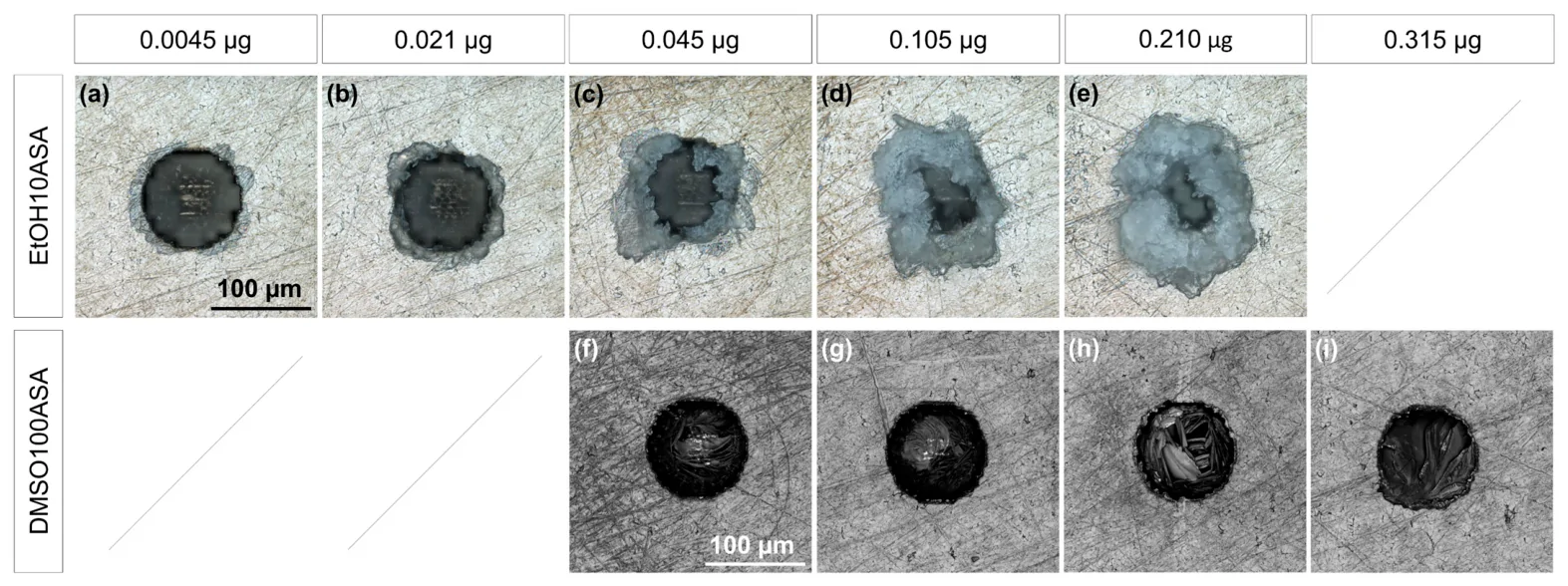
Drug-loaded reservoirs of type 10050 depending on the dispensed mass of model drug ASA and the drug solution that was used. (**a**–**e**) EtOH10ASA: The drug deposit appears crust-like and is deposited at the rim but largely outside of the reservoirs. (**f**–**i**) DMSO100ASA: The drug deposit appears compact inside the reservoirs, featuring plate-like crystalline structures interpenetrating each other. For the highest drug mass of 0.315 µg (**i**), there are minor drug deposits located outside the reservoir.

In the z-dimension, the reservoirs display a cone-like shape, particularly noticeable in the 10050 reservoirs, which have relatively low diameters (Figure 3). As is noticeable in Figure 2(a-4,b-4) and Figure 3, the nominal depth of 50 µm is only approximately achieved. The bottom of a reservoir is not uniformly flat but exhibits an uneven topography, with the deepest region located near the outer areas of the reservoir.

### 3.2. Preparation of Drug Solutions and Wetting Behavior Results

Both drug solutions were successfully prepared via the described procedure. Table 4 shows the results of the contact angle measurements. EtOH10ASA completely wets the steel surface (θ ≈ 0°). DMSO100ASA shows lower wettability than EtOH10ASA; nevertheless, with respect to θ << 90°, it is still significantly wetting the steel surface.

### 3.3. Drug Deposition Results

**Figure 5:**
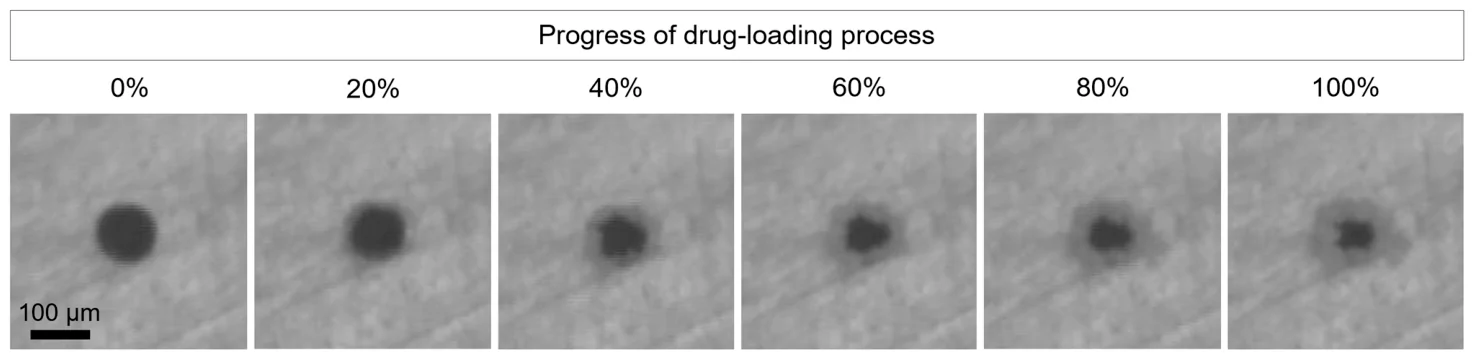
Progress of the drug-loading process of a reservoir of type 10050 when using EtOH10ASA. The dispensed drug mass is 0.105 µg. The images were captured via automated process monitoring via Nano-Plotter 2.1 device target recognition camera. File S4 of the Supplementary Materials provides an animated illustration.

Figure 4 provides an overview of drug-loaded reservoirs of type 10050 depending on the dispensed mass of drug and the utilized drug solution. Please see Figures S1–S9 from File S1 of the Supplementary Materials to assess further microscopy images from replicate experiments. Figure 5 illustrates the progress of drug deposition using the example of a drug mass of 0.105 µg. Additionally, Figure S23 of File S4 of the Supplementary Materials provides an animation of the full progression. The drug deposit appears to “creep” out of the reservoir without any indication of an initial overfilling of the reservoir with the liquid drug solution.

In the case of EtOH10ASA, the drug is deposited mainly outside the reservoirs and is relatively evenly distributed around the rim of the reservoir, and the deposit appears crust-like, which is characteristic of highly branched crystalline structures. The reservoir bottom appears to remain free of drug deposition. This behavior was replicated in all experiments when reservoirs of type 10050 were drug-loaded using EtOH10ASA. In contrast, in the case of DMSO100ASA, the drug deposits appear more compact, with crystalline structures shaped like elongated plates that interpenetrate. In this case, precise drug deposition within the boundaries of the reservoirs was largely achieved. Comparatively minor drug deposits outside the reservoirs were found in one of three experiments for the relatively high drug masses of 0.210 µg and, furthermore, in two of three experiments for the highest drug mass of 0.315 µg.

**Figure 6:**
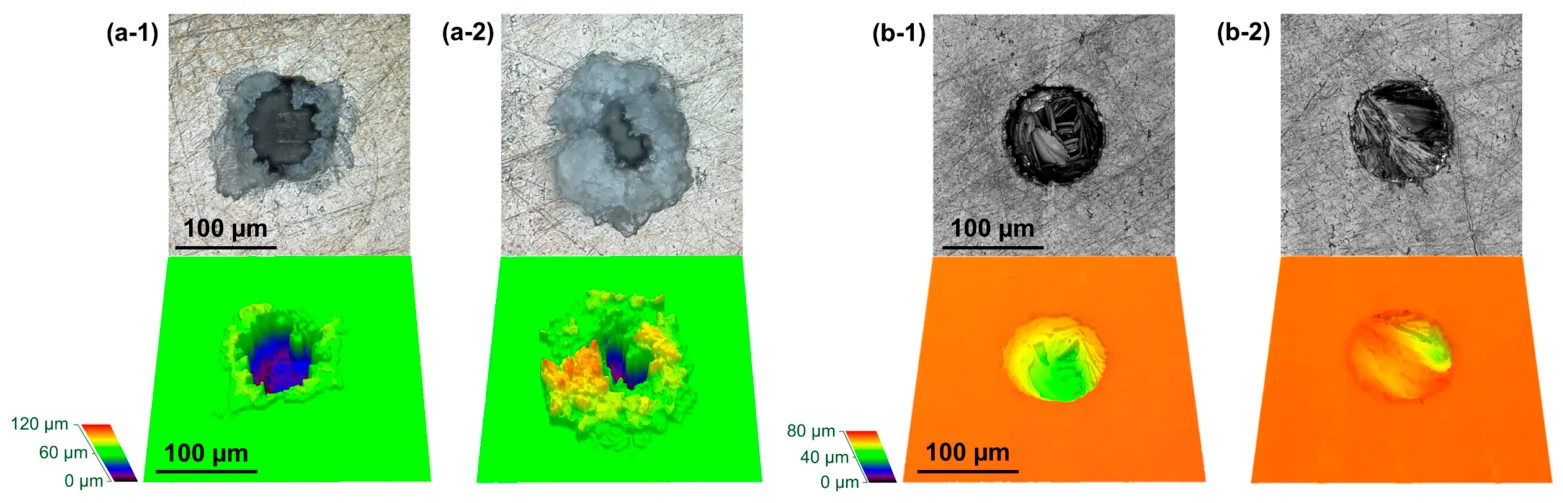
Light microscopy images and three-dimensional imaging of drug-loaded reservoirs of type 10050 obtained by CLSM. The reservoirs were loaded with ASA using different solvents and drug loads: (**a-1**) 0.045 µg ASA with EtOH10ASA, (**a-2**) 0.210 µg ASA with EtOH10ASA, (**b-1**) 0.210 µg ASA with DMSO100ASA, and (**b-2**) 0.315 µg ASA with DMSO100ASA. When EtOH10ASA was applied, the drug deposit expanded in all spatial directions, largely outside the reservoir. In contrast, application of DMSO100ASA resulted in relatively confined deposition within the reservoirs, although the ideal homogeneity was not achieved due to the formation of discrete crystalline structures.

Figure 6 presents exemplary three-dimensional CLSM images of drug-loaded reservoirs of type 10050, with corresponding two-dimensional images shown for reference. Reservoirs loaded with 0.045 µg and 0.210 µg of ASA after dispensing EtOH10ASA are shown, as well as reservoirs loaded with 0.210 µg and 0.315 µg of ASA after dispensing DMSO100ASA. For EtOH10ASA, the three-dimensional visualization clearly demonstrates that the drug deposit spreads and grows in all spatial directions. Although the majority of the deposit forms outside the reservoir, the overall drug volume appears high. At a deposited mass of 0.210 µg ASA, the drug volume would likely exceed the reservoir capacity even if the entire deposit had been confined within the reservoir.

In contrast, when applying DMSO100ASA, the reservoir is filled relatively homogeneously. Nevertheless, the homogeneity of drug distribution within the reservoir seems to be limited by variations in the crystalline structure of the drug deposit. At a drug mass of 0.210 µg, the reservoir’s capacity is not yet fully utilized, whereas at a mass of 0.315 µg, the reservoir appears completely filled. However, due to an inhomogeneous drug distribution, the reservoir is slightly overfilled in some areas and underfilled in others.

**Figure 7:**
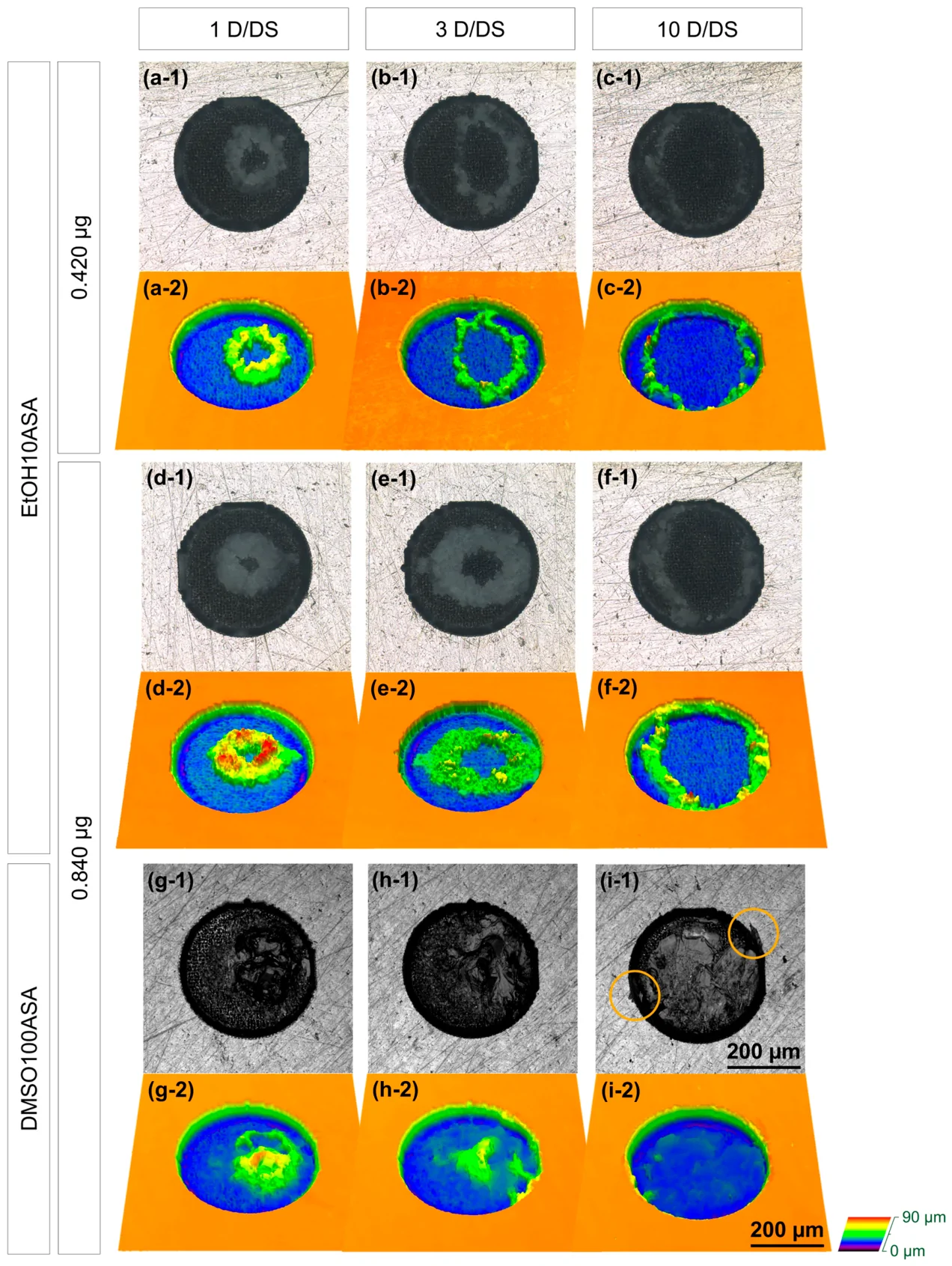
Light microscopy images (**a-1**–**i-1**) and three-dimensional imaging (**a-2**–**i-2**) of drug-loaded reservoirs of type 40050 obtained by CLSM, shown depending on the applied drug solution, deposited mass of drug, and the number of dosed droplets per dispensing step (D/DS). EtOH10ASA resulted in pronounced ring-like drug deposits, whereas such structures were not observed for DMSO100ASA. A higher number of D/DS leads to further expansion of drug deposit and an increased risk of drug deposit extending along the reservoir wall for both drug solutions. In contrast, low D/DS leads to less expansion and higher local accumulation of the drug deposit. But there is a risk of exceeding the reservoir limit in height, especially when utilizing EtOH10ASA (see red areas in (**d-2**)). Since DMSO100ASA provides a more compact drug deposition, it allows for applying higher drug masses (**g-2**). Using a number of 10 D/DS can lead to wall crossings of the drug deposits even when using DMSO100ASA (**i-1**, circled in orange).

Figure 7 provides an overview of drug-loaded reservoirs of type 40050 depending on the used drug solution, the number of jetted droplets per dispensing step, and the deposited mass of drug. Figures S10–S18 in File S1 of the Supplementary Materials show microscopy images from replicate experiments. For EtOH10ASA, ring-like drug deposits are entirely present. The maximum lateral expansion of the ring-like drug deposition and the size of the central area that appears free of drug deposits correlate with the increasing number of droplets per dispensing step. When 10 droplets were applied per dispensing step, the drug deposits spread and climbed up the walls of the reservoirs. In one of five experiments for both relatively high and less high drug masses of 0.840 µg and 0.420 µg, respectively, the drug deposit even extended laterally beyond the reservoir boundaries. Based on the qualitative visual assessment of the microscopic images, the drug-covered area of the reservoir bottom and the height of the drug deposits correlate with the deposited mass of the drug. As a result, the reservoir height may be significantly exceeded when the drug deposition is too highly localized. In particular, this observation was found when loading a relatively high drug mass of 0.840 µg using EtOH10ASA and applying the minimal number of 1 droplet per dispensing step (Figure 7(d-2)).

For DMSO100ASA, no ring-like drug deposition occurs. The drug deposits are distributed relatively homogeneously across the reservoir bottom. Visual assessment of the microscopic images indicates that the size of the covered area seems to correlate with the number of droplets per dispensing step. When 10 droplets are applied per dispensing step, then multiple significant wall crossings of the drug deposits can be observed, as marked in Figure 7(i-1), which was repetitively found in four of five experiments. When only a count of 3 droplets or even of 1 droplet per dispensing step is applied, wall crossings occur only to a very minor extent, in one of five experiments each.

**Figure 8:**
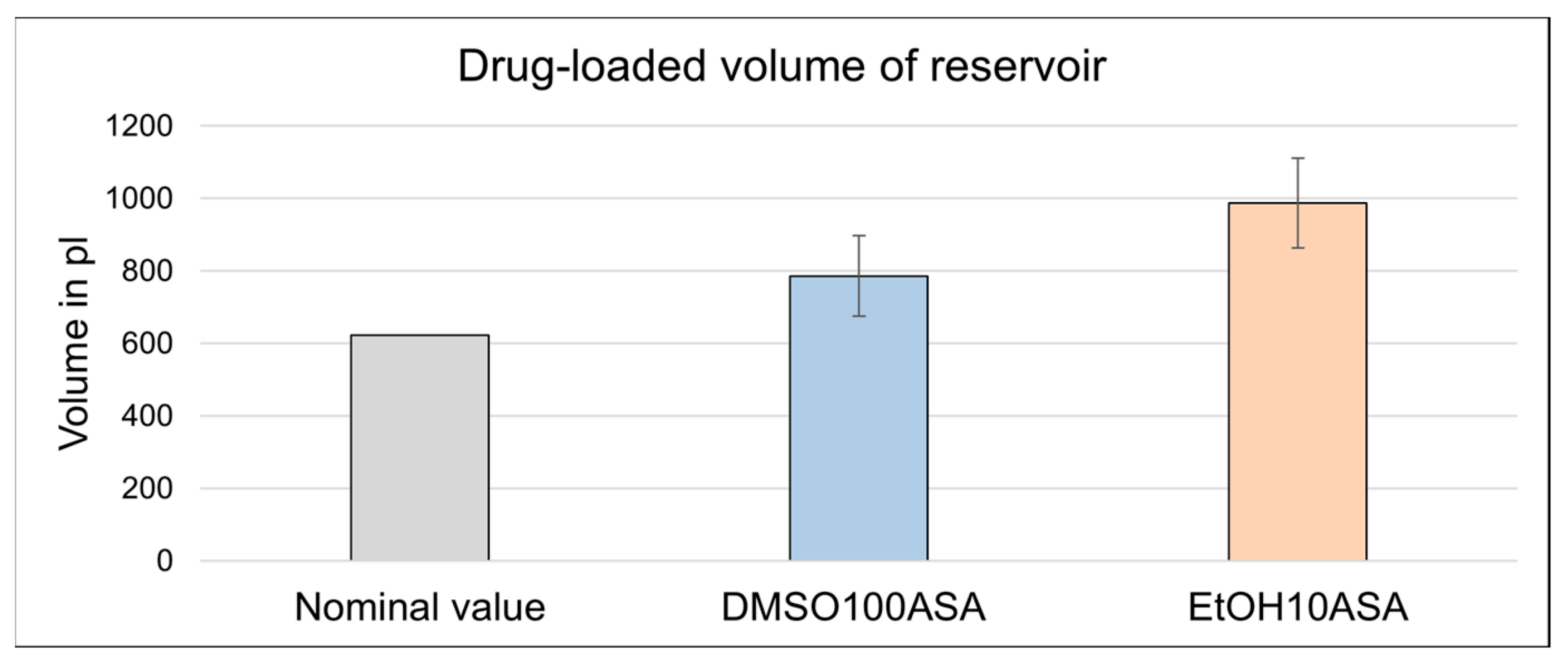
Exemplary comparison of the drug-loaded volume of reservoirs of type 40050. The drug mass of 0.84 µg was loaded using 3 droplets per dispensing step (*n* = 5, mean ± standard deviation).

Figure 8 shows a comparison of the drug-loaded volume for the reservoirs of type 40050 loaded with a drug mass of 0.84 µg using 3 droplets per dosing step. This experiment was selected for exemplary comparison because it achieved the highest positioning precision for both DMSO100ASA and EtOH10ASA, while avoiding distortion of the results due to multiple misloadings. The measurements were derived from CLSM data and are compared to the nominal value that was calculated with a (true) density of ASA of ρ = 1.35 g/cm^3^ [41]. The results indicate that the drug deposits from DMSO100ASA (V = 785 ± 111 pl) require approximately 20% less volume than the drug deposits from EtOH10ASA (V = 987 ± 124 pl). This is consistent with the observation that ASA crystallized from DMSO100ASA more compactly than ASA crystallized from EtOH10ASA. Consequently, the volume of the DMSO100ASA-based drug deposits is in closer agreement with the nominal value (V = 622 pl) based on the true density.

**Figure 9:**
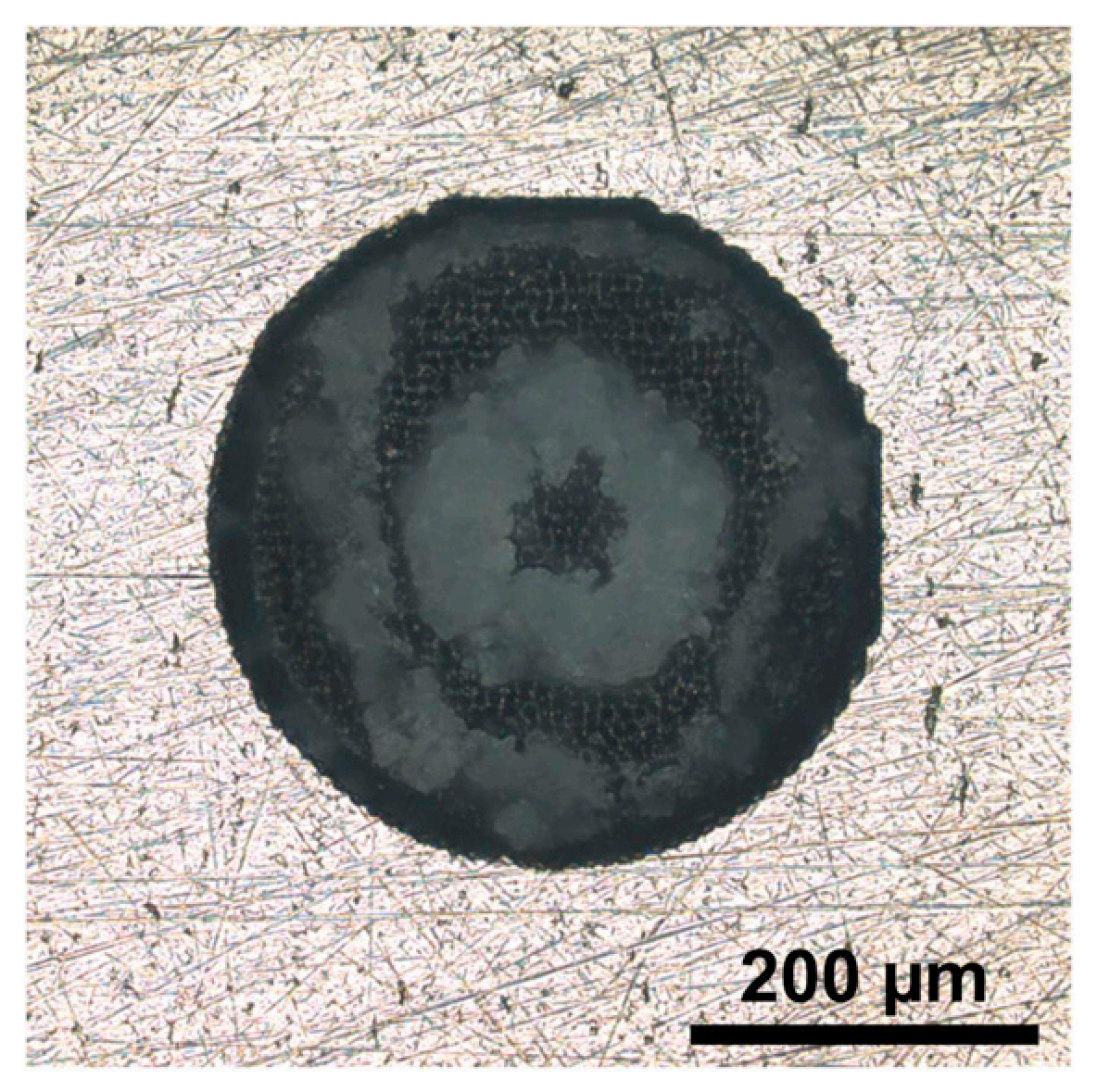
Light microscopy image of a 40050 reservoir loaded with 1.26 µg ASA using EtOH10ASA. As a proof of principle, the drug was deposited using a dispensing strategy that combined different numbers of droplets per dispensing step. The outer ring of the drug deposit contains 0.84 µg of ASA and was formed from 1680 droplets dispensed at 10 droplets per step. The inner ring contains 0.42 µg of ASA and was deposited using 840 droplets dispensed at 1 droplet per step. This dispensing strategy is promising for partial correction of the ring-like, inhomogeneous drug deposition.

The ring-like, inhomogeneous drug deposition from EtOH10ASA can be compensated to some extent by varying the number of jetted droplets per dispensing step. Figure 9 shows an exemplary drug deposit of a total drug mass of 1.26 µg, serving as a proof of principle. It was realized via a combination of 10 and 1 droplets per dispensing step. The outer ring of the drug deposit contains 0.84 µg of ASA, delivered by 1680 droplets at 10 droplets per step. The inner ring contains an additional 0.42 µg of ASA, corresponding to 840 droplets that were subsequently dispensed at 1 droplet per step. Figure S19 in File S1 of the Supplementary Materials shows microscopy images from replicate experiments.

## 4. Discussion

### 4.1. Coffee-Ring Effect

Based on the qualitative evaluation of microscopy images, drug deposition from EtOH10ASA was generally less homogeneous than from DMSO100ASA due to the formation of ring-like drug deposits. The coffee-ring effect is assumed to be the primary cause. In contrast, for DMSO100ASA, no ring-like drug deposits were present at all. It can be assumed that the coffee-ring effect was suppressed for DMSO100ASA, because the low evaporation rate of DMSO favors the growth of fewer but larger ASA crystals. Although crystallization is influenced by multiple factors, a lower evaporation rate can be associated with the formation of larger single crystals and more compact crystalline structures [52,53]. When larger crystals form, they are less likely to deposit at the edge of the evaporating droplet than smaller crystals [20]. In contrast, EtOH10ASA exhibits crust-like crystalline structures likely composed of highly branched, small and fine crystallites. This may be closely related to the high evaporation rate of EtOH. At first glance, these findings might suggest that solutions with low volatility are less prone to the coffee-ring effect. However, in the context of the existing literature, such an interpretation appears overly simplistic, and it is important to consider the type of substance dissolved. Li et al. reported that slower evaporation can intensify the coffee-ring effect in suspensions, as there is more time for the particles to be transported from the center to the edge of the droplet [54], whereas Soltman et al. observed the opposite trend for polymer solutions [55]. The authors found that accelerated droplet evaporation due to heating increased solute transfer to the droplet’s edge. Their results are associated with increased liquid flow from the center to the droplet’s edge due to accelerated evaporation, especially at the droplet’s edge. Such effects will be cross-influenced, for instance, by morphology and size of the particles in solution, or rather suspension [56]. Within this context, crystallizing substances may exhibit behavior distinct from that of non-crystallizing colloidal suspensions due to substance-specific nucleation and crystal growth [20]. For the present results, the differences in the evaporation rates of the applied drug solutions are highly likely to play a crucial role in the intensity of the coffee-ring effect.

### 4.2. Creeping

The widely spread drug deposits observed for EtOH10ASA are associated with pronounced creeping. This is supported by time-resolved investigations of an exemplary drug-loading process and the evaporation of macroscopic droplets of EtOH-based drug solutions, which showed spreading of the drug deposits during droplet evaporation (Figure 5; Files S2–S4 of Supplementary Materials). Furthermore, the crust-like morphology of the drug deposits appears similar to creeping-associated highly branched deposits from salt solutions as shown in [22,23,25]. As a result, creeping was found to be a major factor limiting the precision of drug deposition, in particular when EtOH was applied. This effect is assumed to be the main reason for the inadequate precision of drug deposition in the 10050 reservoirs that was found in all experiments in which EtOH10ASA was applied. The effect is of less relevance for DMSO100ASA. It should be noted that crystallization is influenced by a multitude of factors. Nevertheless, the lower relevance of creeping for DMSO100ASA is plausible given the significant differences in the volatility of EtOH and DMSO. EtOH is highly volatile, whereas DMSO is a low-volatility solvent. Creeping is evaporation-driven [22,23,24]. High evaporation rates can be associated with intense creeping, whereas low evaporation rates correspond to reduced creeping [52]. Generally, at high evaporation rates, the time for crystal growth is limited. As a result, crystal nucleation dominates crystal growth. Consequently, small, fine crystals and highly branched crystalline structures form, which are highly wettable and exhibit intense creeping [23,24]. The low evaporation rate of DMSO leads to more compact structures consisting of larger individual crystals. As a result, creeping is significantly reduced. This is indicated to be the dominant reason for the significantly more precise filling of the reservoir of type 10050 when applying DMSO100ASA.

Furthermore, based on findings from a specifically selected experiment, the more compact crystallization present for DMSO100ASA causes the same amount of drug to require ~20% less volume within the reservoir than when using EtOH10ASA (Figure 8). Nevertheless, the interpenetration of crystalline structures limits the agreement with the calculated nominal value. It should be noted, however, that the nominal value was calculated using the true density of ASA of ρ = 1.35 g/cm^3^. Future work may focus on determining the packing density with respect to the applied individual crystallization conditions, which would allow for a more accurate prediction of the expected values. Moreover, CLSM should be validated as a quantitative method for determining the drug-loaded volume, particularly for crust-like, likely highly branched structures such as those investigated in the present study.

### 4.3. Physicochemical Properties and Wetting Behavior of the Solvents

Further aspects arise when considering the fluid properties of the drug solutions. The solution could be prepared readily because both EtOH and DMSO provide sufficient solubility of ASA [51]. It was found that EtOH10ASA is more wetting than DMSO100ASA. This is in line with the physicochemical properties of the solutions, as EtOH10ASA has a lower surface tension (22.32 ± 0.10 mN/m for EtOH10ASA vs. 42.14 ± 0.44 mN/m for DMSO100ASA) and a lower viscosity (1.13 ± 0.01 mPa·s for EtOH10ASA vs. 2.42 ± 0.06 mPa·s for DMSO100ASA). Moreover, the found contact angles are in accordance with findings for pure EtOH [57,58] and DMSO [59] on steel surfaces. The dissolution of ASA at the applied concentrations does not influence the initial wetting behavior of the solvents on the steel surface significantly. The influence of solvent wetting behavior, surface tension, and viscosity on drug deposition behavior must be considered alongside the dominant role of evaporation rate. Differences in surface tension affect wetting behavior and droplet geometry, thereby influencing evaporation profiles and internal flow fields, with lower surface tension promoting flatter droplets and enhanced edge evaporation. This mechanism plausibly contributes to the stronger coffee-ring effect observed for EtOH10ASA compared to DMSO100ASA. Furthermore, higher solvent viscosity reduces evaporation-driven flow velocities and thus weakens convective solute transport, further contributing to the absence of coffee-ring formation observed for DMSO100ASA.

Similarly, in the context of creeping, the lower surface tension and lower viscosity of EtOH10ASA enhance capillary-driven spreading, and, as a result, promote a more intensive wetting behavior than is the case for DMSO100ASA. These properties facilitate further wetting of the growing crystalline structures that form at the droplet’s triple contact line. Furthermore, intensive wetting increases the droplet evaporation rate [60,61], whereas higher viscosity is associated with a decrease in the droplet evaporation rate [62]. Nevertheless, these effects are assumed to be secondary, because the absolute differences between the solvents in surface tension, viscosity, and wetting behavior are relatively small compared to the pronounced disparity of vapor pressure (0.55 hPa for DMSO vs. 59 hPa for EtOH at T = 20 °C [63,64]). It is likely that the large difference in the evaporation numbers (≈700 for DMSO vs. ≈8.3 for EtOH), or rather the difference in the evaporation rate, is particularly related to the difference in vapor pressure.

The evaporation rate primarily governs the strength of capillary flow, the timing of supersaturation, and crystallization dynamics. The underlying dynamics may be summarized as follows: The high evaporation rate of EtOH leads to rapid supersaturation, particularly at the edge of the droplet (three-phase contact line), where solvent evaporation is typically highest [20]. To dissipate this supersaturation, crystal nucleation dominates over crystal growth, leading to highly branched crystalline structures that occupy a comparatively large area. The low viscosity promotes fluid transport towards the droplet edge, thereby intensifying the coffee-ring effect. Furthermore, the low surface tension as well as low viscosity promote continued wetting of crystallizing structures that grow and spread at the droplet’s triple contact line, thereby intensifying creeping. In contrast, the low evaporation rate of DMSO provides sufficient time for supersaturation to dissipate through crystal growth. Local gradients in drug concentration within the solution are reduced by diffusion, thereby limiting excessive crystal nucleation. As a result, fewer but larger single crystals and more compact crystalline structures form, which mitigates creeping intensity and suppresses the coffee-ring effect. Moreover, the higher viscosity and higher surface tension reduce fluid transport towards the droplet edge, which further suppresses both phenomena.

Taken together, the findings suggest that the pronounced disparity in evaporation rates between EtOH and DMSO plays a significant role in drug deposition. This interpretation is reasonable because the evaporation rate strongly influences the crystallization process and the morphology of the growing ASA crystals. However, it should be noted that the evaporation rate was not isolated as the sole influencing parameter affecting drug deposition. The investigated drug solutions were based on different solvent systems with individual solvent–solute interactions. Although a low evaporation rate may promote crystal growth, generally, larger crystals can also result from other factors promoting slower supersaturation development and lower nucleation rates. This leaves considerable scope for further investigations employing other solvents.

### 4.4. Model Drug

The model drug ASA, with its low molecular mass of 180.16 g/mol [41] and relatively less complex molecular structure containing an aromatic ring and a carboxyl group [30,47], is expected to exhibit a high tendency toward crystallization. This is consistent with general knowledge on the crystallization behavior of drugs [45,46]. The pronounced creeping intensity during crystallization from high-volatility EtOH-based drug solution is likely related to this characteristic. Despite rapid evaporation, ASA undergoes crystallization, which is a prerequisite for the creeping phenomenon. As discussed above, the rapid evaporation of EtOH promotes crystal nucleation over crystal growth, resulting in pronounced creeping.

Other small-molecule drugs with relatively high molecular masses just below 1000 g/mol and comparatively complex molecular structures may exhibit different deposition behavior due to their distinct crystallization kinetics. If such substances do not crystallize completely but instead remain predominantly in an amorphous state, they are unlikely to be affected significantly by creeping. The same applies to drugs of high molecular weight, such as proteins and peptides, which generally exhibit little to no tendency to crystallize and are therefore also unlikely to undergo creeping.

The coffee-ring effect can occur for both crystallizing and non-crystallizing substances [19,20]. However, particle morphology has a significant influence on the resulting deposition pattern [56]. It is worth noting that particles in crystallizing systems undergo continuous change due to nucleation and the progress of crystal growth, whereas this is not the case for non-crystallizing systems, such as most colloidal suspensions [20].

It is important to note that the present study does not include investigations regarding impairment of drug stability and drug purity, for instance from residual solvent content or hydrolysis from hygroscopicity. Adequate drug stability and purity should be ensured through targeted investigations tailored to the intended implant application and the drug of interest. Moreover, a well-defined control of ambient humidity is advisable not only in the context of drug stability but for influencing evaporation [65] and, therefore, crystallization behavior as well. Based on the assessment by microscopy in the present work, the crystallization patterns were generally reproducible. However, it should be noted that a more stringent control of ambient humidity will most likely be required to obtain more detailed and robust results.

### 4.5. Dispensing Strategy

Moreover, precision and homogeneity of the drug deposition depend on the number of jetted droplets per dispensing step. Applying a low number of droplets, that is, a low deposited volume per dispensing step, generally resulted in drug deposits that were less widely spread across the surface area. In the literature, it is well established that smaller droplet volumes lead to higher resolution in inkjet printing [66]. This is particularly beneficial for depositing multiple drug depots within a single reservoir, as it allows different drug depots to be placed adjacent to one another. The application of a low number of droplets per dispensing step to achieve precisely localized drug deposition remains relevant even under conditions of intense creeping. In this context, advantage may be taken of the fact that secondary creeping becomes dominant over primary creeping. The results may suggest that, over the course of the drug-loading process, lateral spreading associated with primary creeping becomes less dominant, and the drug deposit increasingly expands in multiple spatial directions (Figure S22 of File S2 and also File S3 of the Supplementary Materials provide further supporting results). However, for optimal precision, it is essential that drug accumulations do not locally exceed the height limits of the reservoir, as shown in Figure 7(d-2). In contrast, a higher number of droplets per dispensing step may enable a more efficient utilization of the available surface area, that is, the reservoir capacity (demonstrated for DMSO100ASA, Figure 7(i-1)). Care must be taken, however, as excessively high droplet counts may compromise lateral deposition precision. Likely driven by creeping, the drug deposit can extend beyond the reservoir wall.

Furthermore, it was demonstrated that the combination of different numbers of jetted droplets per dispensing step can be an effective strategy to compensate for the coffee-ring effect. The sequential application of dispensing steps with high droplet count, followed by steps with low droplet count, enabled the incorporation of multiple drug depots within a single reservoir (Figure 9). This approach is especially beneficial for efficient utilization of the reservoir capacity when the coffee-ring effect cannot be avoided. In this context, it should be noted that the coffee-ring effect is commonly considered to be disadvantageous [67,68]. Nevertheless, the present results indicate that this phenomenon can be deliberately exploited to load a reservoir with multiple depots of different drugs.

### 4.6. Processing Time

When considering the influence of solvent evaporation rate and dispensing strategy, it should be noted that these parameters can substantially affect the overall processing time of drug loading. This aspect must be considered when evaluating the economic feasibility of the drug-loading process. With an evaporation number of approximately 700 for DMSO compared to an evaporation number of 8.3 for ethanol, DMSO requires roughly an 84-fold longer evaporation time. In addition, the use of multiple dispensing steps multiplies the individual drying times.

Conversely, DMSO100ASA exhibits a tenfold higher drug concentration than EtOH10ASA, reducing the number of required dispensing steps by a factor of ten. Nevertheless, the different drying times applied in this work (Table 2) resulted in significant differences in total process time. For example, when dispensing the relatively high mass of 0.84 µg ASA with a count of 1 droplet per dispensing step, the process time for EtOH10ASA was approximately 5040 s, i.e., 84 min (1680 steps with a drying time of 3 s per step). Additionally, the printhead was washed for 30 s every 4 min. Twenty-one washing cycles were performed with a total duration of 10.5 min. Considering a further 7 min of printhead priming (about 20 s per washing cycle), the total process time was approximately 102 min. In contrast, for DMSO100ASA, the total process time was approximately 1008 min, i.e., 16.8 h (168 steps with a drying time of 6 min per step, with negligible time required for initial printhead priming and no extra washing cycles needed). In particular, the long total process time when using DMSO may be critical in terms of drug stability, which requires further investigation.

Optimizing drying times to a minimum was beyond the scope of this study. Based on the findings in [69], a further reduction of initial drying times may be possible. In the context of drug loading of implants, however, it is important to note that additional, potentially time-consuming post-processing steps for residual solvent removal may be required. Generally, implant applications with multiple reservoirs offer potential for reducing total process time through process-step parallelization.

### 4.7. Laser-Drilled Micro-Reservoirs

Finally, the applied micro-reservoir manufacturing approach, based on laser drilling using ultrashort laser pulses, offers promising opportunities to tailor implant surface properties for optimized drug deposition. In general, the high repeatability and high throughput potential of micro-reservoir manufacturing, as well as the sharpness of the reservoirs’ contours, are in accordance with the known advantages of laser drilling with ultrashort laser pulses [40]. Due to the extremely short laser pulses, the energy transfer rate into the material is very high. This allows a rapid ablation with strongly reduced heat diffusion. Typically, this leads to the minimization of recast layers or burr formation, which is advantageous for the precision of material ablation, and enables high dimensional precision and surface quality [39,70,71,72]. Moreover, the technique is of relevance for a very wide range of materials, including metals, ceramics, glass, and polymers [70,73,74].

The applied laser drilling process can be optimized to improve the homogeneity of energy distribution, thereby mitigating taper and irregularities at the reservoir bottom. Multiple options, such as optimized laser scanning strategies [75,76,77] as well as optimized laser parameters such as laser fluence [75] and laser polarization [78], can be employed.

Ultrashort laser pulse technology also enables modification of surface wetting properties through surface laser structuring [79,80]. This could be exploited for more precise and homogeneous drug deposition. The widespread drug deposits observed for both DMSO100ASA and EtOH10ASA suggest pronounced wetting, contact-line pinning, and high drug adhesion at the relatively inhomogeneous reservoir bottom surface. Restricting initial droplet spreading, as well as reducing contact-line pinning and the adhesion between the drug and the surface, may decrease the lateral extent of crystalline deposits and modify or suppress coffee-ring formation, as shown for superhydrophobic surfaces [53]. Ultrashort-pulse laser structuring is a relevant approach for generating tailored wetting states, including superhydrophobic surfaces [80]. However, such surface modification must be balanced against the need for sufficient drug adhesion, which may be required to retain the drug within the reservoir and bind it to the implant surface. The geometric confinement provided by the reservoir itself may offer additional flexibility in designing the surface properties of the reservoir bottom. This aspect should be addressed in future studies.

## 5. Conclusions

The present study investigates the influence of evaporation-driven phenomena, namely the coffee-ring effect and creeping, on the precision and homogeneity of inkjet-based deposition of a crystallizing drug into surface-embedded micro-reservoirs. The role of the physicochemical properties of the inkjet-printed drug solution, with a focus on the evaporation behavior, was examined. In addition, the effect of the number of jetted droplets per dispensing step was studied. The presented results provide guidance for selecting suitable solvents and inkjet process parameters for localized drug deposition, particularly for polymer-free drug loading of implant surfaces.

The coffee-ring effect and creeping can impair drug deposition quality. The extent of these effects depends strongly on the drug solution employed. A highly volatile EtOH-based ASA solution (c = 10 g/L) exhibited pronounced coffee-ring formation and creeping, resulting in reduced precision and homogeneity. In contrast, a DMSO-based ASA solution (c = 100 g/L), characterized by low volatility, showed more compact ASA crystallization, no coffee-ring effect, and only minor creeping. Consequently, the DMSO-based ASA solution enabled more precise and more homogeneous drug deposition, allowing reliable loading of smaller reservoirs compared to EtOH-based ASA solution.

A low droplet count per dispensing step is associated with high precision in localized drug deposition; however, attention should be given to both the lateral spread and the vertical accumulation. As demonstrated in a proof-of-principle experiment, combining different droplet counts may improve the homogeneity of drug distribution affected by a pronounced coffee-ring effect. Moreover, it is worth noting that such a dispensing strategy could exploit the coffee-ring effect advantageously by enabling the deposition of multiple drug depots within a single reservoir.

In this study, the differences in evaporation rate of the solvents are considered a key factor underlying the observed results. However, it should be noted that crystallization is a highly multifactorial process. Future research should aim to better isolate and control individual factors influencing drug deposition, or rather, drug crystallization behavior, such as the evaporation rate or the concentration of drug in solution, thereby allowing a more precise evaluation of their specific impact. To this end, focusing on a single solvent system and a specific drug chosen for a desired implant application may be appropriate. Additionally, a promising field for further investigations is surface engineering for adjusting the wetting characteristics and the surface adhesion of crystalline structures. Laser structuring methods are promising for such an approach.

Finally, it should be emphasized that this study is limited to investigations of drug deposition processes using model systems. The presented results can only be considered indicative of general trends, as the evaluation was conducted primarily in a qualitative manner. Quantitative assessments of drug-loaded volumes were based on specifically selected experiments. The applicability of the presented findings to other drug formulations intended for specific implant applications remains to be addressed. Once a specific implant application is targeted, quantitative requirements for homogeneity and precision should be clearly defined and assessed. Moreover, particular attention should be given in future research to post-processing steps such as residual solvent removal and the functional performance of drug-loaded implants, including drug stability, drug release, and biological response.

## Figures and Tables

**Table 1:** Overview of drug loading experiments (reservoirs 10050: *n* = 3, reservoirs 40050: *n* = 5).

Type of Micro-Reservoir	Drug Mass in µg	Total Number of Dispensed Droplets		Droplet Count per Dispensing Step		
		EtOH10ASA	DMSO100ASA			
10050	0.0045	9	-	1		
	0.0210	42	-	1		
	0.0450	90	9	1		
	0.1050	210	21	1		
	0.2100	420	42	1		
	0.3150	-	63	1		
40050	0.4200	840 *	-	1	3	10
	0.8400	1680	168 *	1	3	10
	1.2600	2520	-	First 10 (i) and subsequently 1 (ii); Procedural details: (i) 10 droplets per step, 1680 droplets in total, initial deposited mass 0.84 µg (ii) 1 droplet per step, 840 droplets in total, additional deposited mass 0.42 µg		

* When applying a droplet count per dispensing step of 10, there are 17 dispensing steps. The final dispensing step delivers only 8 droplets.

**Table 2:** Drying times in relation to the drug solution and the applied droplet count per dispensing step. A single droplet had a nominal volume of 50 pl.

Drug Solution	Droplet Count per Dispensing Step	Drying Time per Dispensing Step
EtOH10ASA	1	≈3 s
	3	≈3 s
	10	≈3 s
DMSO100ASA	1	≈6 min
	3	≈25 min
	10	≈35 min

**Table 3:** Averaged entrance diameter and volume of the laser-drilled reservoirs obtained by CLSM (*n* = 20).

Type of Micro-Reservoir	Averaged Entrance Diameter ± Standard Deviation	Averaged Volume ± Standard Deviation
10050	103 ± 1 µm	316 ± 7 pl
40050	411 ± 3 µm	5677 ± 300 pl

**Table 4:** Averaged results of contact angle measurements (*n* = 3) of drug solutions on surface of EN 1.4404 (AISI 316L) steel specimen (Ra ≈ 0.1 µm).

Drug Solution	Contact Angle θ ± Standard Deviation
EtOH10ASA	Complete wetting (θ ≈ 0°)
DMSO100ASA	35.4 ± 3.4

## Data Availability

The original contributions presented in this study are included in the article and Supplementary Materials. Further inquiries may be directed to the corresponding author.

## Supplementary Materials

The following supporting information can be downloaded at: https://www.mdpi.com/article/10.3390/jfb17080420/s1, File S1: Overview of microscopy images of drug-loaded micro-reservoirs from replicate experiments, File S2: Illustrated comparison of the evaporation of macroscopic droplets of EtOH10ASA and EtOH100ASA, File S3: Video of sequential evaporation of seven droplets of EtOH100ASA, and File S4: Animation of the drug-loading process when using EtOH10ASA.
