# Supplementary material for: Inkjet Printing of Drugs into Surface-Embedded Micro-Reservoirs for Drug-Releasing Implants: Influence of Solvent Properties on Deposition Behavior
Source: J Funct Biomater. 2026 Aug 20;17(8):420. doi: 10.3390/jfb17080420 (PMC13514584; doi:10.3390/jfb17080420)
Supplement: Supplementary file 1 [file jfb-17-00420-s001.zip › Suppl. Mat. File S1 - Overview of microscopy images of drug-loaded micro-reservoirs for replicate experiments.pdf]

Supplementary Materials, File S1:

Overview of microscopy images of drug-loaded micro-reservoirs from replicate experiments

Purpose: This overview of microscopy images acquired during the drug loading experiments (Figures S1–S19) is intended to provide a qualitative assessment of reproducibility.

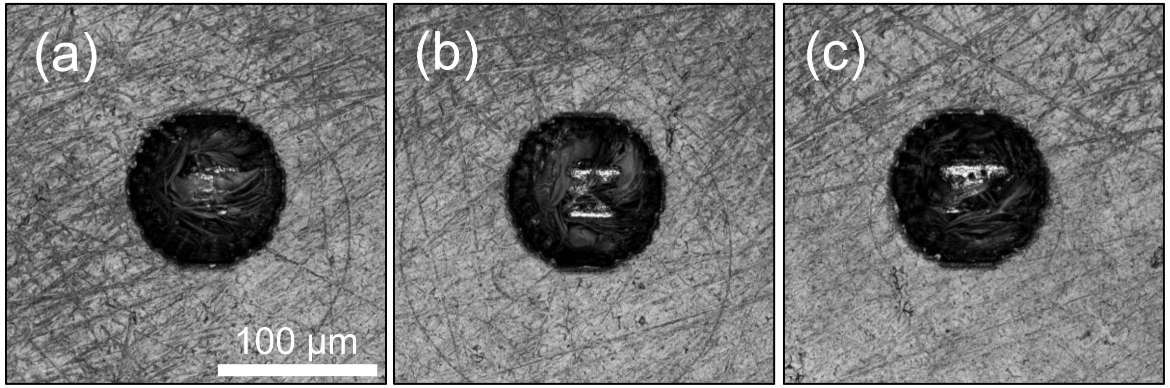

Figure S1: DMSO100ASA / 10050 / 0.045 µg / 1 *

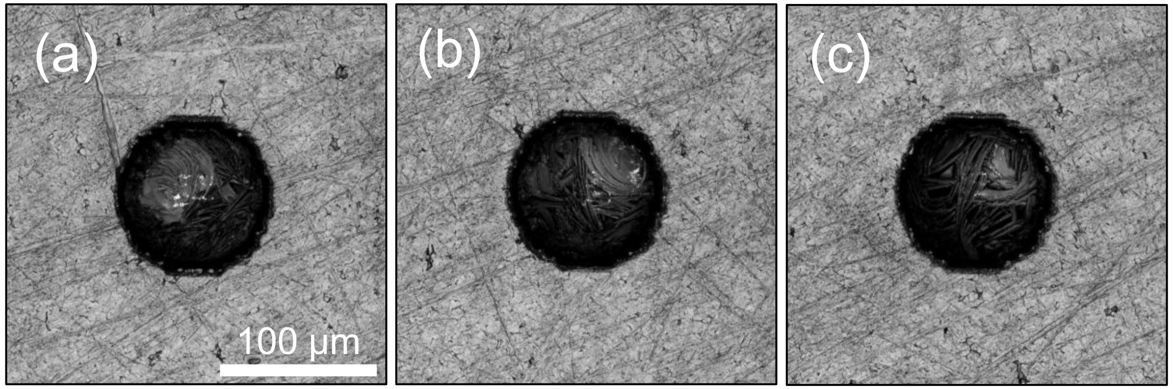

Figure S2: DMSO100ASA / 10050 / 0.105 µg / 1

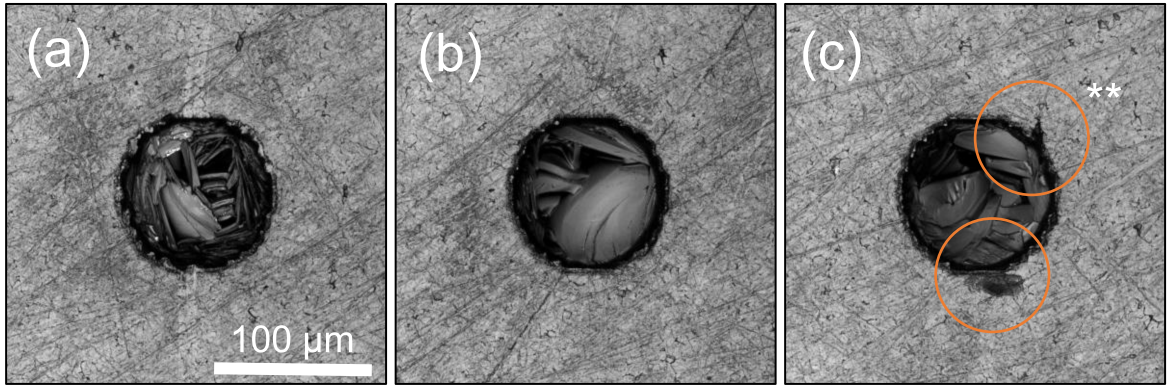

Figure S3: DMSO100ASA / 10050 / 0.210 µg / 1

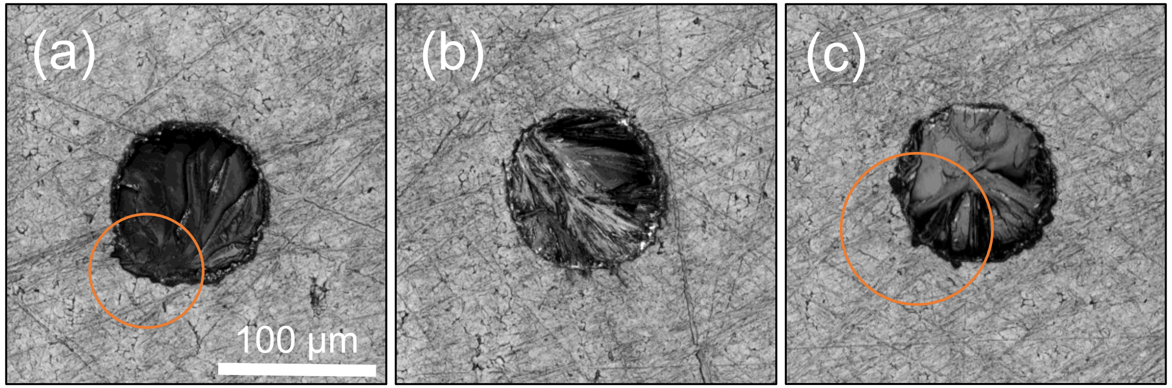

Figure S4: DMSO100ASA / 10050 / 0.315 µg / 1

* Designation: Applied drug solution / Type of micro-reservoir / Drug mass / Droplet count per dispensing step

** Circled in orange: Imprecisions in drug deposition (drug deposit has exceeded the reservoir wall)

Each subfigure (identified by a letter) represents the result of an individual replicate experiment.0

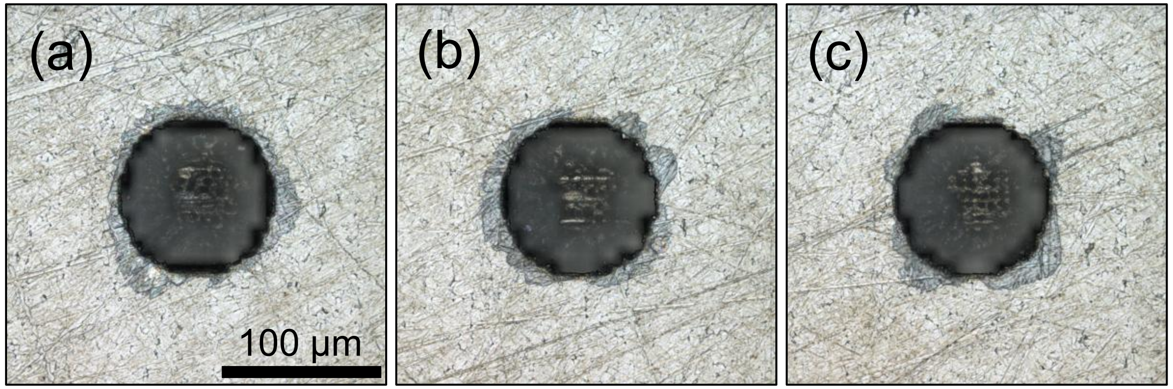

Figure S5: EtOH10ASA / 10050 / 0.0045 µg / 1

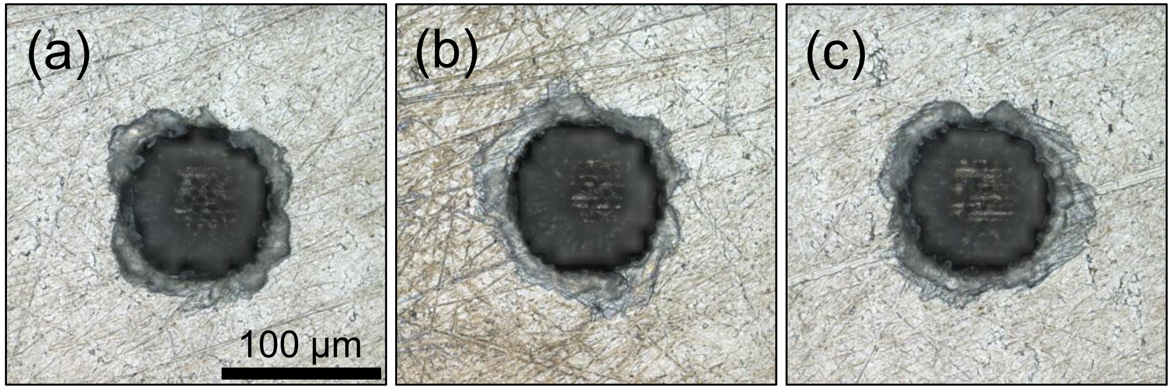

Figure S6: EtOH10ASA / 10050 / 0.021 µg / 1

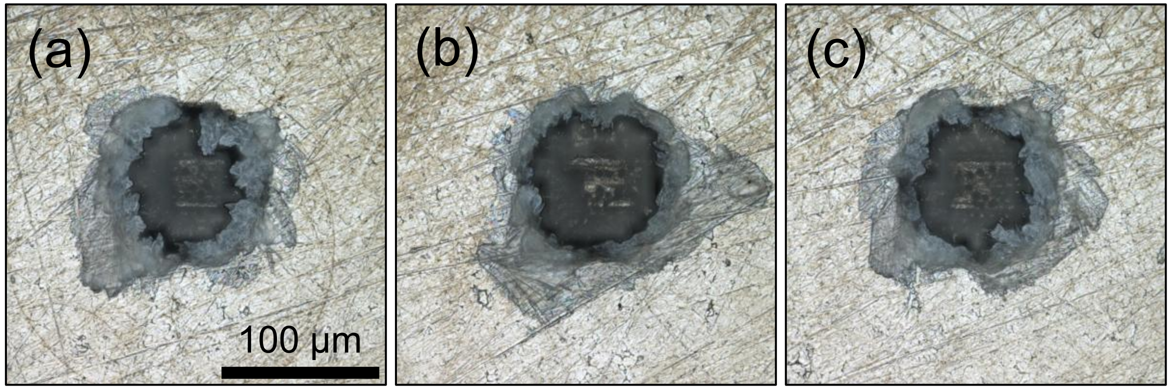

Figure S7: EtOH10ASA / 10050 / 0.045 µg / 1

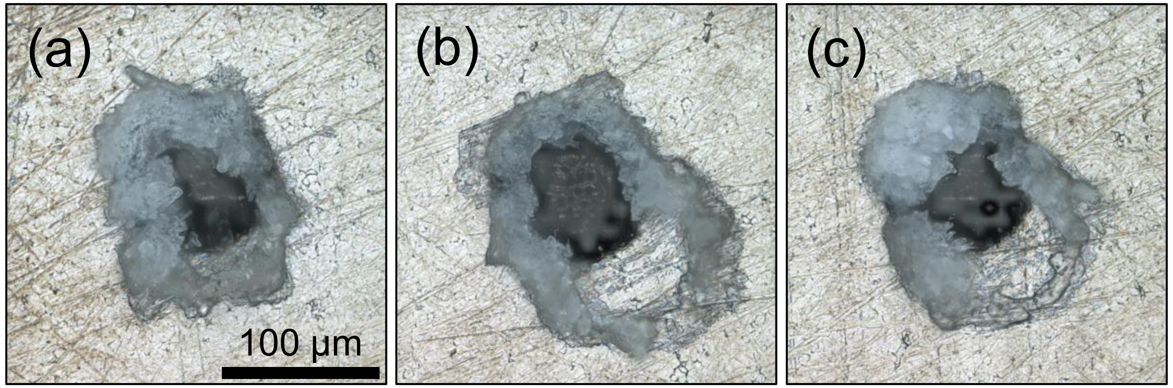

Figure S8: EtOH10ASA / 10050 / 0.105 µg / 1

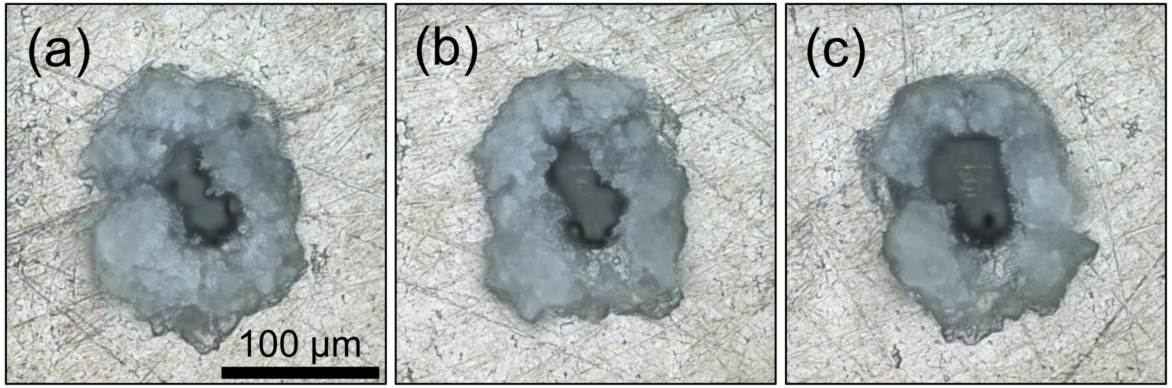

Figure S9: EtOH10ASA / 10050 / 0.210 µg / 1

Remark: Imprecisions in drug deposition (the drug deposit exceeded the reservoir wall) were observed in every experiment and were therefore not marked individually.

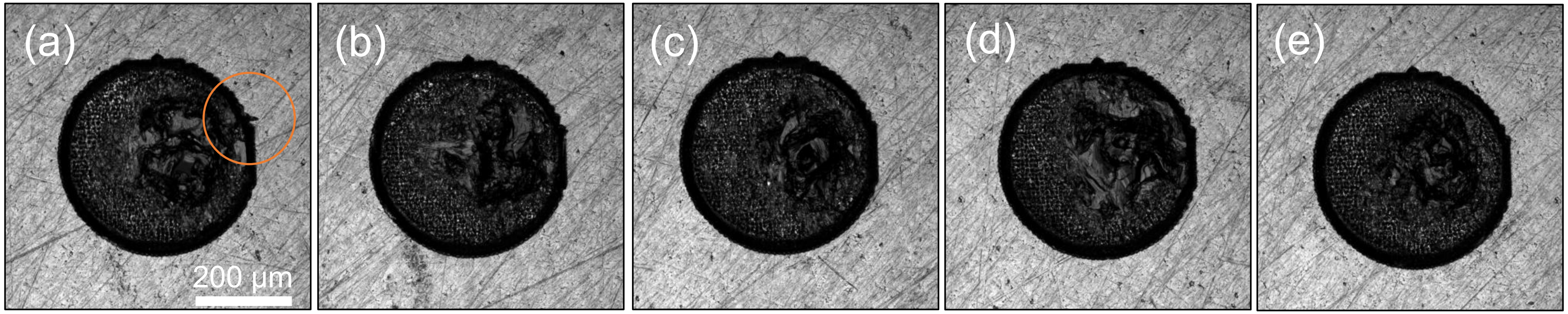

Figure S10: DMSO100ASA / 40050 / 0.84 µg / 1

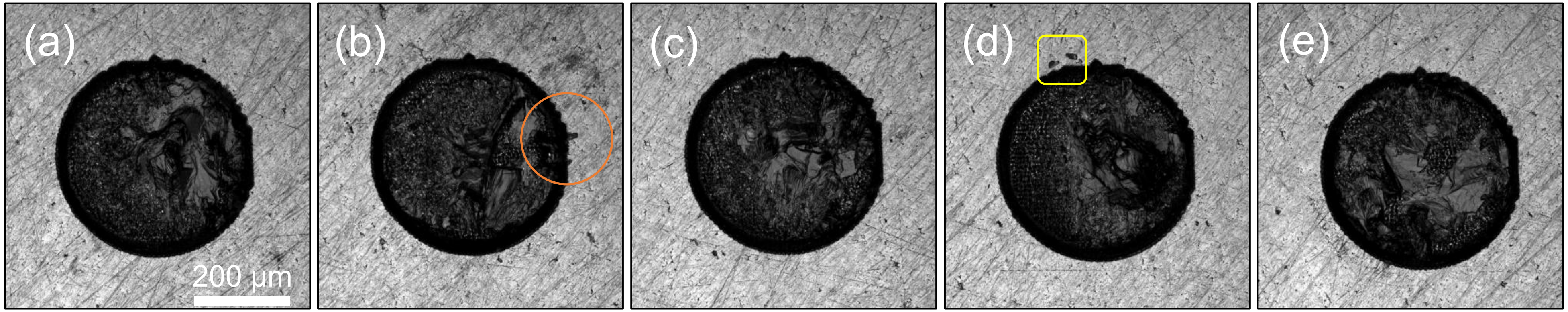

Figure S11: DMSO100ASA / 40050 / 0.84 µg / 3

Framed in yellow: Imprecision likely caused by satellite droplets (outlier case)

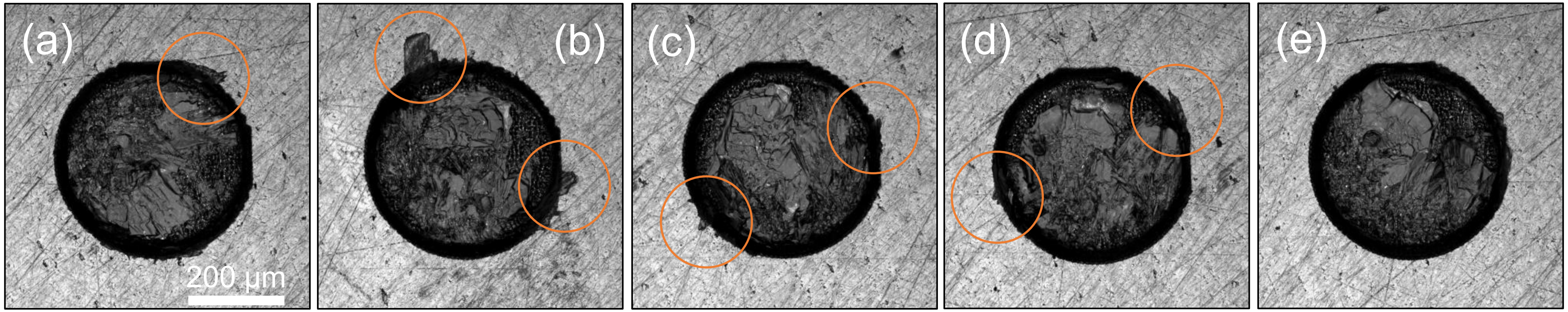

Figure S12: DMSO100ASA / 40050 / 0.84 µg / 10

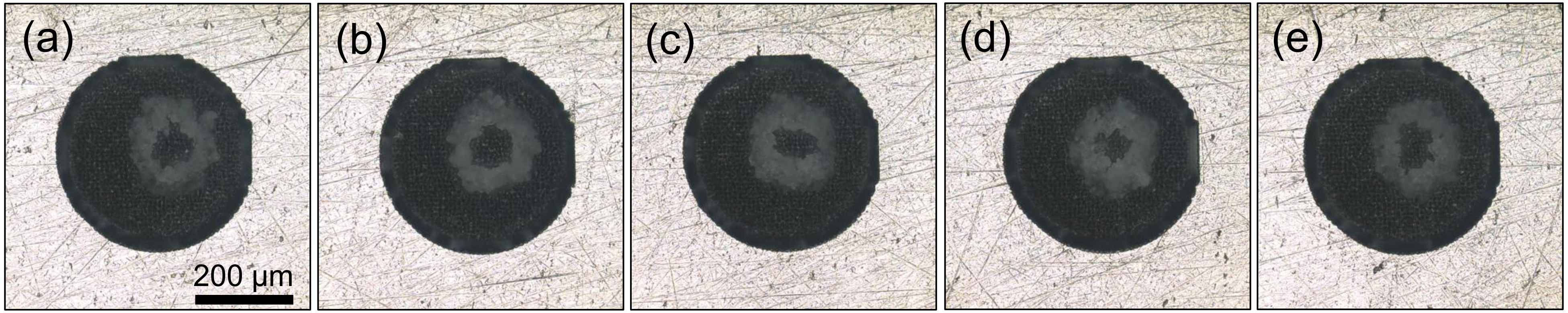

Figure S13: EtOH10ASA / 40050 / 0.42 µg / 1

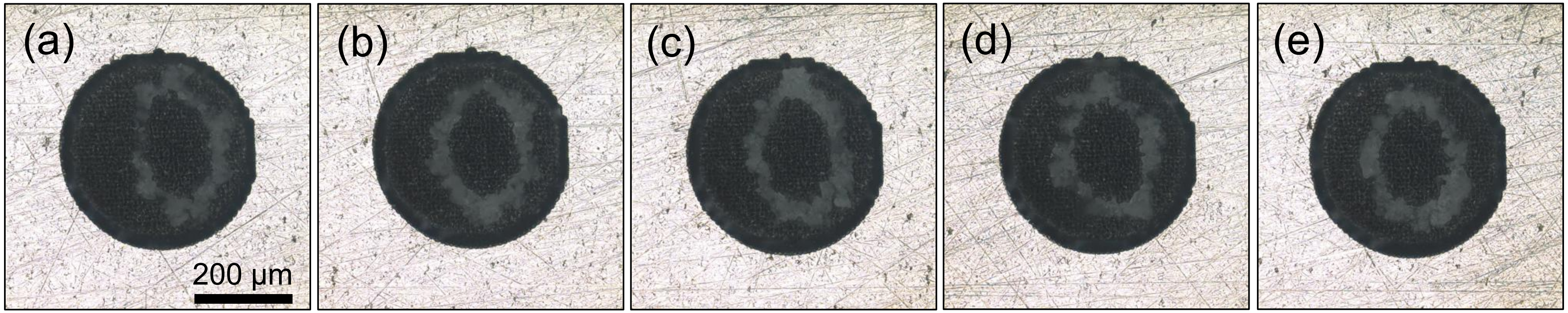

Figure S14: EtOH10ASA / 40050 / 0.42 µg / 3

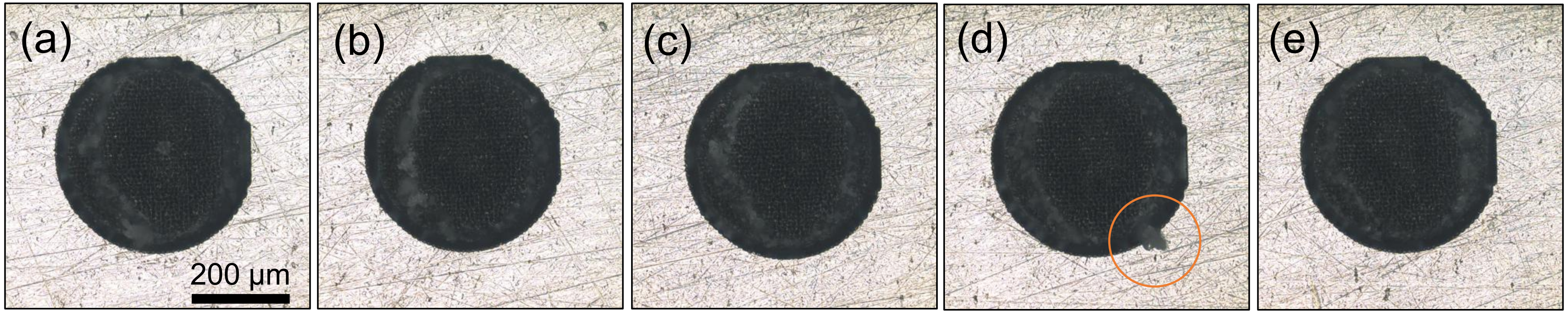

Figure S15: EtOH10ASA / 40050 / 0.42 µg / 10

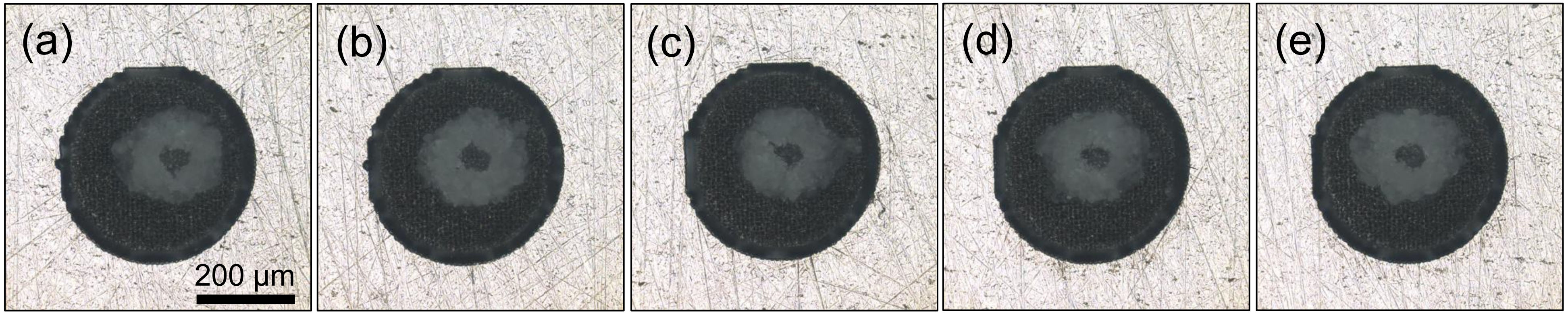

Figure S16: EtOH10ASA / 40050 / 0.84 µg / 1

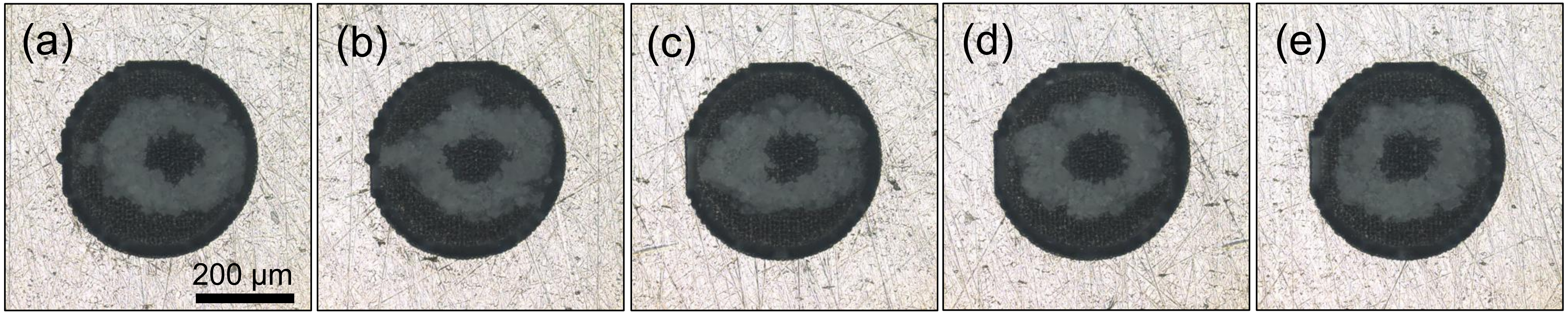

Figure S17: EtOH10ASA / 40050 / 0.84 µg / 3

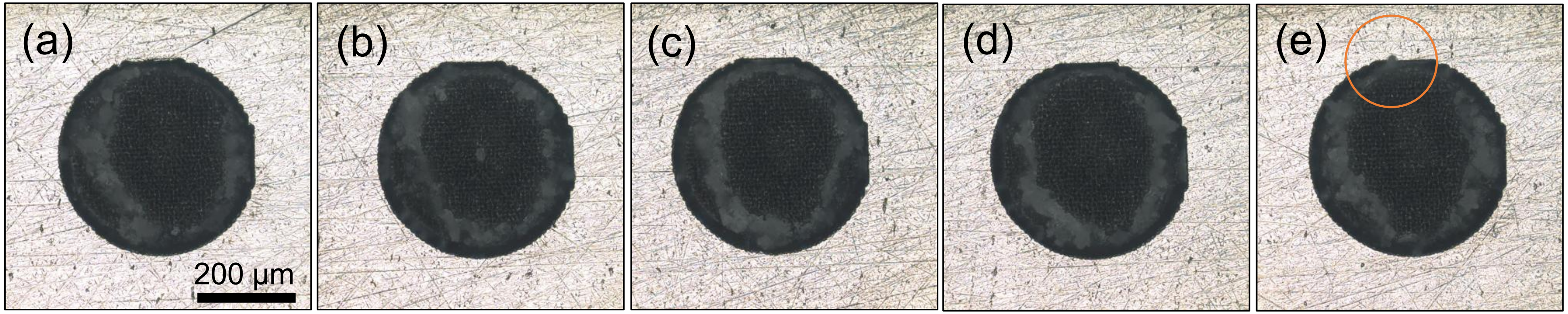

Figure S18: EtOH10ASA / 40050 / 0.84 µg / 10

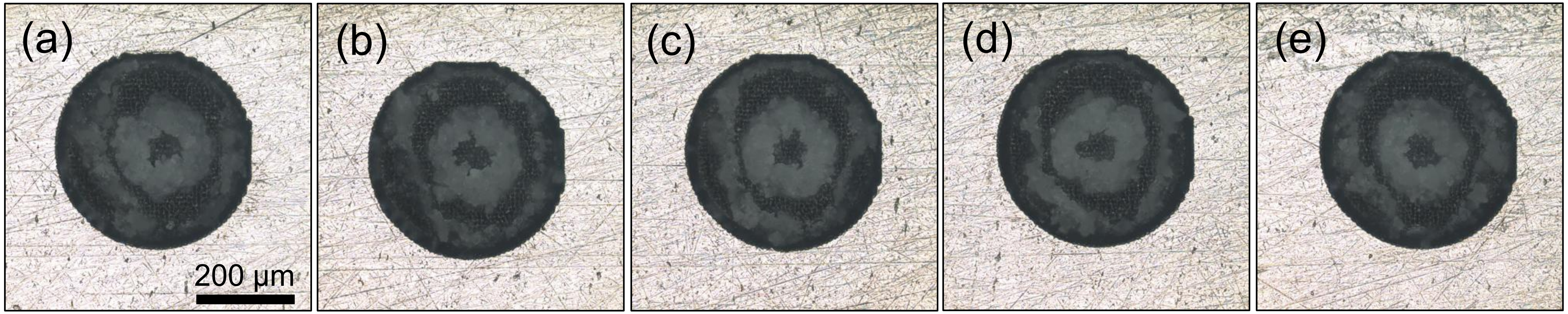

Figure S19: EtOH10ASA / 40050 / 1.26 µg / Combination of 10 (for 0.84 µg, initial) and 1 (for 0.42 µg, subsequent) droplets per dispensing step
