# Supplementary material for: Inkjet Printing of Drugs into Surface-Embedded Micro-Reservoirs for Drug-Releasing Implants: Influence of Solvent Properties on Deposition Behavior
Source: J Funct Biomater. 2026 Aug 20;17(8):420. doi: 10.3390/jfb17080420 (PMC13514584; doi:10.3390/jfb17080420)
Supplement: Supplementary file 1 [file jfb-17-00420-s001.zip › Suppl. Mat. File S2 - Illustrated comparison of the evaporation of macroscopic droplets of EtOH10ASA and EtOH100ASA.pdf]

Supplementary Materials, File S2:

Illustrated comparison of the evaporation of macroscopic droplets of EtOH10ASA and EtOH100ASA

Purpose: Comparative investigations on the evaporation process of macroscopic droplets (V = 6 µl) of EtOH-based drug solutions of concentrations of c = 100 g/L (EtOH100ASA) and c = 10 g/L (EtOH10ASA), see Figure S20 and Figure S21. Providing further details on the lateral spreading of drug deposits after sequential drying of multiple droplets, exemplified by EtOH100ASA (Figure S22).

Remark 1: EtOH100ASA is not a feasible option for inkjet-based drug deposition into micro-reservoirs due to insufficient printability

Remark 2: File S3 of the Supplementary Materials provides a video of the sequential evaporation of seven droplets of EtOH100ASA.

Materials and Methods: Positioning of a single droplet (V = 6 µl) on a stainless steel disc (d = 6 mm; without laser-drilled micro-reservoirs). Observation of progress of evaporation utilizing the camera of the contact angle and contour analysis system OCA 40 Micro (DataPhysics Instruments GmbH, Filderstadt, Germany).

Results: Please see Figure S20 and Figure S21. Principally, EtOH100ASA and EtOH10ASA show a comparable evaporation as well as drug deposition behavior. Droplet spreading continues during the drying process after initial wetting of the surface. This behavior is associated with creeping of crystalline structures. As a result, the drug deposit exceeds the edges of the steel disc. After evaporation, the ASA drug deposits show a crust-like morphology. Moreover, the drug deposition shows the coffee ring effect. EtOH100ASA shows more widespread drug deposition than that observed for EtOH10ASA, which is associated with significantly higher content of drug per droplet.

Please see further Figure S22 and also File S3 (video) of the Supplementary Materials. It can be seen that the lateral spreading decreases over the course of the process. This indicates that secondary creeping becomes dominant over primary creeping.

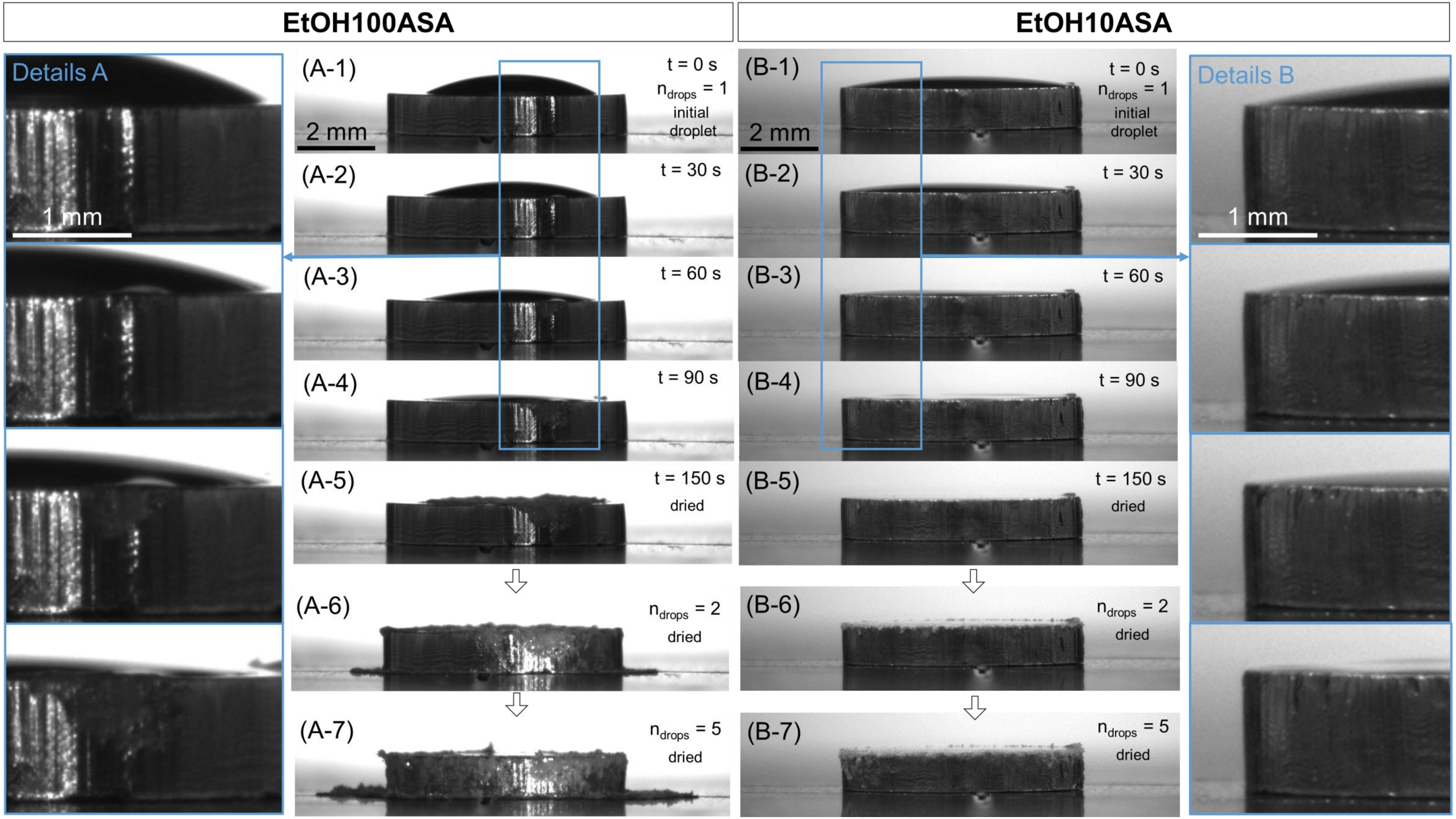

Figure S20: Evaporation of droplets (V = 6 µl) of EtOH100ASA (**A-1** to **A-7** and **Details A**) and EtOH10ASA (**B-1** to **B-7** and **Details B**) on steel discs (d = 6 mm).

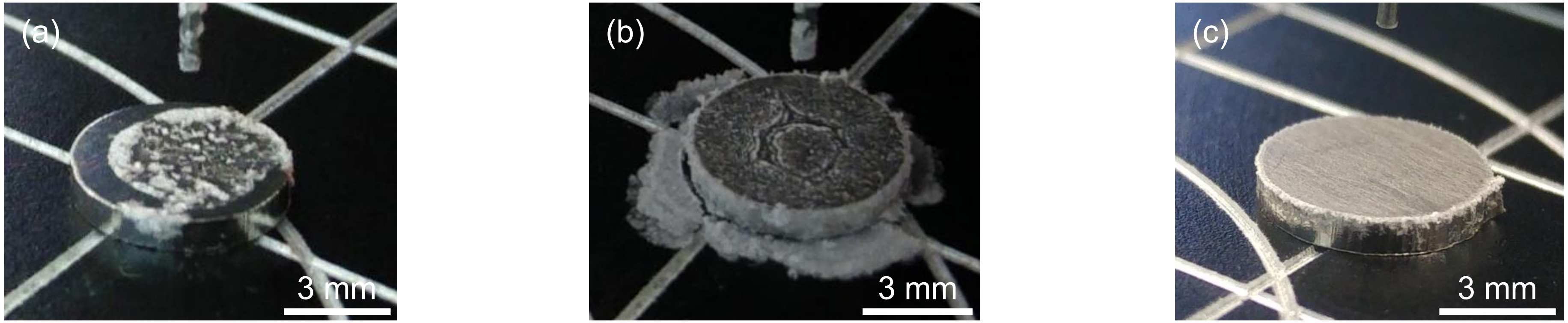

Figure S21: Photographs of drug deposits on steel discs after drying of different numbers of droplets. **(a)** Drug deposit of ASA after drying of single droplet (V = 6 µl) of highly concentrated EtOH100ASA drug solution. Edge-crossings of drug deposit caused by creeping. Crust-like drug deposit due to highly-branched crystalline structures. Coffee ring effect is present. **(b)** Drug deposit of ASA after sequential drying of four droplets (V = 6 µl each) of EtOH100ASA drug solution. The drug deposit spread further. **(c)** Drug deposit of ASA after sequential drying of five droplets (V = 6 µl) of less concentrated EtOH10ASA drug solution. Edge-crossings of drug deposit caused by creeping. Crust-like drug deposit due to highly-branched crystalline structures. The coffee ring effect is present.

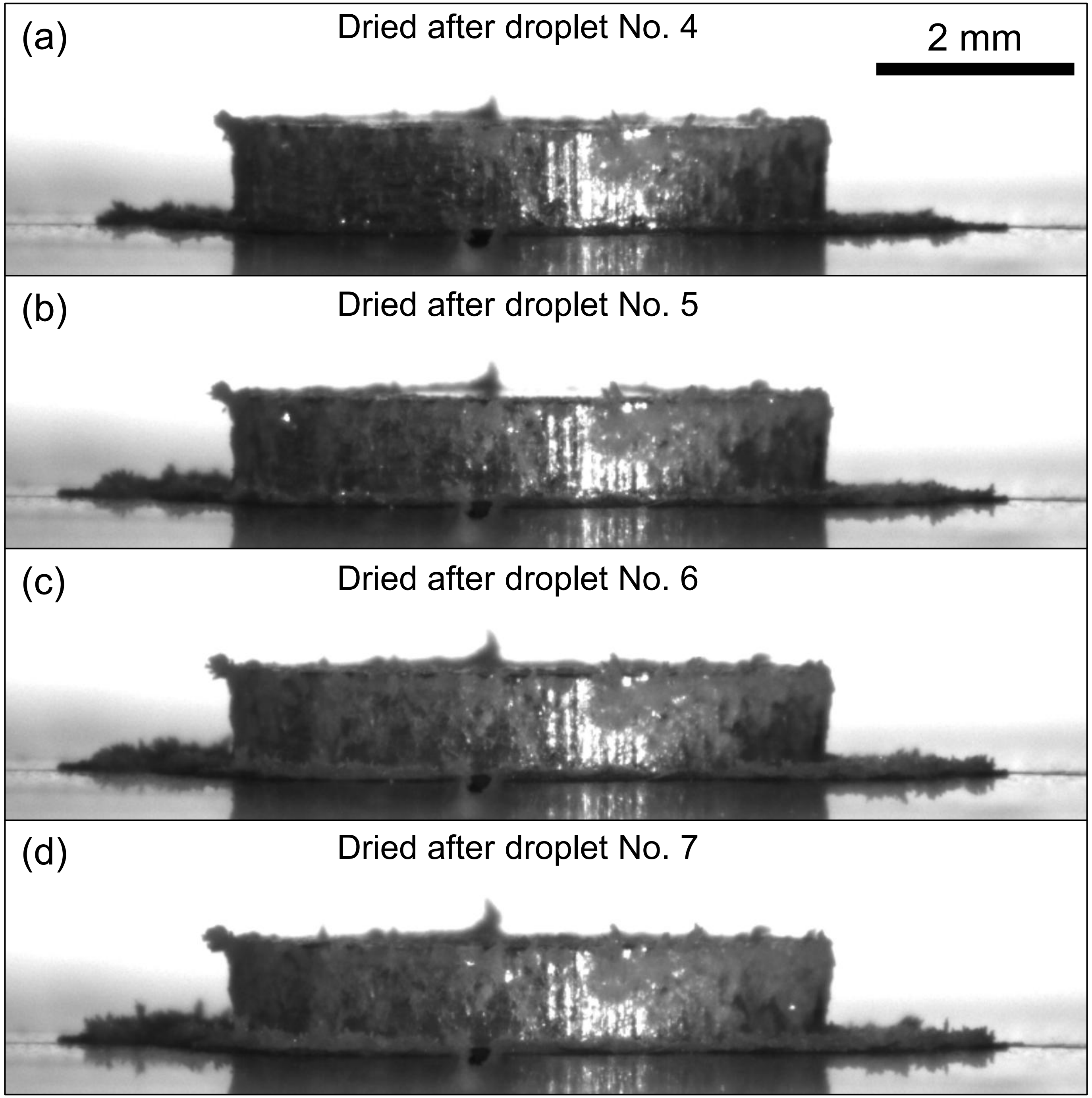

Figure S22:
Dried state of drug deposit after multiple sequentially dried droplets (V = 6 µl each) of EtOH100ASA. **(a)** Dried after droplet number 4. **(b)** Dried after droplet number 5. **(c)** Dried after droplet number 6. **(d)** Dried after droplet number 7.

The lateral spreading of the drug deposit decreases because secondary creeping becomes increasingly dominant relative to primary creeping over the course of the experiment.

Please note: File S3 of the Supplementary Material provides a video of the sequential evaporation of seven droplets of EtOH100ASA.
